# Supplementary material for: Monitoring transmission intensity of trachoma with serology
Source: Nat Commun. 2023 Jun 5;14:3269. doi: 10.1038/s41467-023-38940-5 (PMC10241377; doi:10.1038/s41467-023-38940-5)
Supplement: Supplementary file 3 — Description of Additional Supplementary Files [file 41467_2023_38940_MOESM3_ESM.pdf]

Description of Additional Supplementary Files

File Name: Supplementary Data 1
Description: Sensitivity and specificity to identify clusters with C. trachomatis infection by seroprevalence threshold among 1-9 year olds.

File Name: Supplementary Data 2
Description: Sensitivity and specificity to identify clusters with C. trachomatis infection by seroconversion rate threshold among 1-9 year olds.

File Name: Supplementary Data 3
Description: Sensitivity and specificity to identify clusters with C. trachomatis infection by seroprevalence threshold among 1-5 year olds.

File Name: Supplementary Data 4
Description: Sensitivity and specificity to identify clusters with C. trachomatis infection by seroconversion rate threshold among 1-5 year olds.
