# Supplementary material for: Monitoring transmission intensity of trachoma with serology
Source: Nat Commun. 2023 Jun 5;14:3269. doi: 10.1038/s41467-023-38940-5 (PMC10241377; doi:10.1038/s41467-023-38940-5)
Supplement: Supplementary file 5 — Reporting Summary [file 41467_2023_38940_MOESM5_ESM.pdf]

nature portfolio

Corresponding author(s): Benjamin F. Arnold

Last updated by author(s): May 15, 2023

Reporting Summary

Nature Portfolio wishes to improve the reproducibility of the work that we publish. This form provides structure for consistency and transparency in reporting. For further information on Nature Portfolio policies, see our Editorial Policies and the Editorial Policy Checklist.

Statistics

For all statistical analyses, confirm that the following items are present in the figure legend, table legend, main text, or Methods section.

n/a	Confirmed	
☐	☒	The exact sample size (*n*) for each experimental group/condition, given as a discrete number and unit of measurement
☐	☒	A statement on whether measurements were taken from distinct samples or whether the same sample was measured repeatedly
☒	☐	The statistical test(s) used AND whether they are one- or two-sided<br>*Only common tests should be described solely by name; describe more complex techniques in the Methods section.*
☐	☒	A description of all covariates tested
☐	☒	A description of any assumptions or corrections, such as tests of normality and adjustment for multiple comparisons
☐	☒	A full description of the statistical parameters including central tendency (e.g. means) or other basic estimates (e.g. regression coefficient) AND variation (e.g. standard deviation) or associated estimates of uncertainty (e.g. confidence intervals)
☒	☐	For null hypothesis testing, the test statistic (e.g. *F*, *t*, *r*) with confidence intervals, effect sizes, degrees of freedom and *P* value noted<br>*Give P values as exact values whenever suitable.*
☒	☐	For Bayesian analysis, information on the choice of priors and Markov chain Monte Carlo settings
☒	☐	For hierarchical and complex designs, identification of the appropriate level for tests and full reporting of outcomes
☐	☒	Estimates of effect sizes (e.g. Cohen's *d*, Pearson's *r*), indicating how they were calculated

Our web collection on statistics for biologists contains articles on many of the points above.

Software and code

Policy information about availability of computer code

Data collection	No data collection software was used in this secondary analysis of existing, primary data.
Data analysis	R version 4.2.2 (2022-10-21, "Innocent and Trusting") was used for this analysis. Open source code and instructions to reproduce all analyses are available in the Open Science Framework repository, https://osf.io/e6j5a/ .

For manuscripts utilizing custom algorithms or software that are central to the research but not yet described in published literature, software must be made available to editors and reviewers. We strongly encourage code deposition in a community repository (e.g. GitHub). See the Nature Portfolio guidelines for submitting code & software for further information.

Data

Policy information about availability of data

All manuscripts must include a data availability statement. This statement should provide the following information, where applicable:

- Accession codes, unique identifiers, or web links for publicly available datasets
- A description of any restrictions on data availability
- For clinical datasets or third party data, please ensure that the statement adheres to our policy

De-identified data to reproduce this work are publicly available without restriction in the Open Science Framework repository, https://osf.io/e6j5a/ .

Research involving human participants, their data, or biological material

Policy information about studies with human participants or human data. See also policy information about sex, gender (identity/presentation), and sexual orientation and race, ethnicity and racism.

Reporting on sex and gender	Analyses pooled information across sex and gender. Current trachoma elimination programs do not target treatment or intervention by sex or gender, only by age. Analyses and summaries reflect the current surveillance approach by global trachoma programs (all children ages 1-9 years old).
Reporting on race, ethnicity, or other socially relevant groupings	N/A
Population characteristics	The study included children ages 1-9 years old from rural communities in five countries in Africa.
Recruitment	Most contributing studies were population-based samples (as indicated in Figure 1 of the paper). Populations from randomized controlled trials were sampled and recruited with a goal of enrolling representative populations. In all cases, as describe in the following sentences, the trials included a representative sample of communities and then included either random samples of children from communities or all children in a community (complete census), limiting the possibility of selection bias to influence the results. In the WUHA and TAITU trials (Ethiopia 2018), communities that were representative of the study populations were enrolled in the trials and then children ages 1-9y (and 1-5y for PCR testing) were randomly sampled from enrolled communities . In the MORDOR trial (Niger 2018) a sample of 30 communities were randomly selected among more than 600 in the Dosso region as part of a larger mortality trial. Within each of the 30 communities, all children ages 0-5y were enrolled in specimen collection. In the PRET trial (Niger 2013) included 48 communities of which 24 were randomly selected to include dried blood spot collection. Within the 24 communities, a random sample of 50 children ages 1-5y old were identified for specimen collection. In the Kongwa trial (Tanzania 2013), 96 balozis (neighborhoods) were identified from 8 distinct communities. Within each balozi, a random sample of 20 children ages 1-9y were selected to participate in the specimen collection for the trial.
Ethics oversight	The secondary analysis protocol was reviewed and approved by the Institutional Review Board at the University of California, San Francisco (Protocol #20-33198). All primary data that contributed to the analysis was collected after obtaining informed consent from all participants or their guardians under separate, local human subjects research protocols in accordance with the Declaration of Helsinki.

Note that full information on the approval of the study protocol must also be provided in the manuscript.

Field-specific reporting

Please select the one below that is the best fit for your research. If you are not sure, read the appropriate sections before making your selection.

☐ Life sciences ☒ Behavioural & social sciences ☐ Ecological, evolutionary & environmental sciences

For a reference copy of the document with all sections, see nature.com/documents/nr-reporting-summary-flat.pdf

Behavioural & social sciences study design

All studies must disclose on these points even when the disclosure is negative.

Study description	This quantitative study is a pooled analysis of 14 cross-sectional, population-based surveys.
Research sample	The study included 19,811 children ages 1-9 years old from 459 rural communities in five countries in Africa (Ethiopia, Malawi, Morocco, Niger, Tanzania). Original sample sizes were dictated by the individual studies that contributed data to this pooled analysis. We included all available data with two restrictions described below in Data exclusions.
Sampling strategy	Most contributing studies were population-based samples (as indicated in Figure 1 of the paper, and Supplementary Table 1). Populations from randomized controlled trials were also sampled and recruited with a goal of enrolling representative populations. Sample sizes for surveillance studies were guided by standard sampling designs specified by Tropical Data for trachoma elimination surveys based on clinical signs of trachoma among children ages 1 to 9 years old. Typically, this included 25-30 communities per evaluation unit (often a district), and within each community up to 40 children ages 1-9 were randomly sampled. Dried blood spot specimen collection was added to existing clinical surveys. In randomized controlled trials that contributed, sample sizes were originally determined to study the effect of delivering or stopping mass distribution of azithromycin on trachoma or Chlamydia trachomatis infection. Supplementary Table 1 includes an inventory of contributing studies along with their primary citations for study-specific details. Since each, individual study was sufficiently powered to study trachoma indicators, this pooled analysis had sufficient sample size to make comparisons between separate studies. As described in the Research sample (above), we limited the analysis to communities with at least 15 children measured to ensure that we would have reasonable estimates of community-level means.
Data collection	In this secondary analysis of existing data, we did not use data collection instruments. Original studies used electronic data collection methods, typically with custom software that also included barcode scanners for specimen collection and tracking.
Timing	Studies collected primary data between May 2013 and March 2019. The span of years reflects different timing for the original studies and surveys.

Data exclusions	We made two primary data exclusions in this study. First, the randomized controlled trials were longitudinal and included multiple measurements over several years (WUHA, TAITU, PRET, Kongwa2013, MORDOR). In these cases, we included only the most recent year of data collection that also included PCR infection to simplify the interpretation in the case of changing transmission conditions and make the data more comparable to population-based, cross-sectional surveillance surveys. This led to the exclusion of 4,225 (WUHA), 2,510 (Kongwa2013) and 3,895 (MORDOR) children. Blood samples were only collected in a single year in TAITU and PRET so this restriction did not lead to exclusions.<br><br>Second, we excluded clusters from the analysis if they included fewer than 15 children with measurements. We made this second exclusion since several of the analysis relied on cluster-level summaries, and we were concerned that clusters with <15 measurements would have unreliable (noisy) estimates. We described these exclusions in the Methods: "Due to changing transmission and/or control interventions in studies with repeated cross-sectional data (WUHA in Wag Hemra, Ethiopia; Kongwa, Tanzania from 2012-2015; MORDOR in Dosso, Niger), we included only the most recent year of measurements with serology, and PCR measurements if available, for each population. We excluded clusters with fewer than 15 children measured to ensure sufficient information to estimate cluster level means (n=22 clusters excluded, reduced from 481 to 459)."<br><br>The code that makes these exclusions, including some figures showing the rationale, is here: https://osf.io/g3uq8 with notebook here: https://osf.io/fde36<br><br>Note: the full samples have been made available as part of the Source Data for this paper -- see the link to the harmonized datasets in the Open Science Framework. https://osf.io/ykjc4/
Non-participation	This was a secondary analysis of existing data so no participants were directly involved in the study.
Randomization	Five studies from randomized controlled trials contributed data to this analysis, but randomly allocated treatment was not a component of the present analyses. Supplementary Information Table 1 includes detailed citations for the RCTs for additional information. The analyses focused on methodology for estimating seroconversion using age-seroprevalence data and comparisons with other measures of Chlamydia trachomatis infection. Where relevant, we stratified results by important population characteristics (i.e., age, recent mass distribution of azithromycin) to clarify interpretation of the surveillance data.

Reporting for specific materials, systems and methods

We require information from authors about some types of materials, experimental systems and methods used in many studies. Here, indicate whether each material, system or method listed is relevant to your study. If you are not sure if a list item applies to your research, read the appropriate section before selecting a response.

Materials & experimental systems

n/a	Involved in the study
☒	☐ Antibodies
☒	☐ Eukaryotic cell lines
☒	☐ Palaeontology and archaeology
☒	☐ Animals and other organisms
☒	☐ Clinical data
☒	☐ Dual use research of concern
☒	☐ Plants

Methods

n/a	Involved in the study
☒	☐ ChIP-seq
☒	☐ Flow cytometry
☒	☐ MRI-based neuroimaging
