# Supplementary material for: Monitoring transmission intensity of trachoma with serology
Source: Nat Commun. 2023 Jun 5;14:3269. doi: 10.1038/s41467-023-38940-5 (PMC10241377; doi:10.1038/s41467-023-38940-5)
Supplement: Supplementary file 6 — Source Data [file 41467_2023_38940_MOESM6_ESM.zip › trachoma_serology_public_data_cluster_codebook.html]

Trachoma serology cluster-level public data codebook


Code 

- Show All Code
- Hide All Code

# Trachoma serology cluster-level public data codebook

#### updated: 2023-04-07 18:25:34

#### authors: Pearl Ante-Testard (pearl.ante@ucsf.edu) and Ben Arnold (ben.arnold@ucsf.edu)

# Summary

Here, you will find the codebook for the file `trachoma_serology_public_data_cluster_v2` (cluster-level data). There are two formats for each dataset, comma separated values (`.csv`) and R dataset (`.rds`). The contents are the same, but the `.rds` file includes additional encodings, such as factor labels and levels.

Note that there is a companion dataset with individual-level information, named `trachoma_serology_public_data_indiv_v2`. The individual level dataset does not include some data that were only available at the cluster level (and not at the individual level): PRET2013 (PCR and TF/TI), Kongwa2013 (PCR), and TCC-Ethiopia2017 (TF/TI). Therefore, this dataset augments the individual level file, but in most cases the data were simply aggregated (summed or averaged) at the cluster level.

These data were harmonized and made publicly available under the NIH-funded study: *Seroepidemiology of trachoma for the elimination endgame* R01-AI158884.

The R Markdown script that created this codebook is `05-codebook-trachoma-serology-cluster.Rmd` which is called from the R script `05-codebook-trachoma-serology.R`.

The codes below are based on Ruben Arslan’s article on the “codebook” package: https://journals.sagepub.com/doi/full/10.1177/2515245919838783.

```
library(here)
source(here("R/0-config.R"))

# cluster dataset
codebook_data_cluster <- readRDS(file = paste0(box_data_path, "/public-", data_version, "/trachoma_serology_public_data_cluster_",data_version,".rds"))

# to import an SPSS file from the same folder uncomment and edit the line below
# codebook_data <- rio::import("mydata.sav")
# for Stata
# codebook_data <- rio::import("mydata.dta")
# for CSV
# codebook_data <- rio::import("mydata.csv") (R. Arslan)
```

# trachoma\_serology\_public\_data\_cluster\_v2

```
# "omit the following lines, if your missing values are already properly labelled" (R. Arslan)
codebook_data_cluster <- detect_missing(codebook_data_cluster,
    only_labelled = FALSE, # "only labelled values are autodetected as
                                   # missing
    negative_values_are_missing = FALSE, # negative values are missing values
    ninety_nine_problems = TRUE,   # 99/999 are missing values, if they
                                   # are more than 5 MAD from the median" (R. Arslan)
    )

# labelling variables

var_label(codebook_data_cluster) <- list(study_id = "Study ID", country = "Country", district = "District",
                                         eu = "Evaluation unit", eu_desc = "Evaluation unit description",
                                         cluster_id_public = "Public cluster ID", 
                                         year = "Survey year", location_year_name = "Location and year",
                                         mda = "MDA administration within the previous year", 
                                         
                                         pcr_n_pos = "No. of PCR infections detected",
                                         pcr_n_tested = "No. of individuals tested for infection by PCR",

                                         tf_n_pos = "No. of individuals positive for TF", 
                                         tf_n_tested = "No. of individuals tested for TF",
                                         ti_n_pos = "No. of individuals positive for TI",
                                         ti_n_tested = "No. of individuals tested for TI",
                                         pgp3_n_pos = "No. of positive Pgp3 response",
                                         pgp3_n_tested = "No. of individuals tested for Pgp3",
                                         ct694_n_pos = "No. of positive CT694 response",
                                         ct694_n_tested = "No. of individuals tested for CT694",
                                         pgp3ct694_n_pos = "No. Pgp3 and CT694 seropositives",
                                         pgp3ct694_n_tested = "No. of individuals tested for Pgp3 and CT694",
                                         pgp3_mean = "Mean Pgp3 IgG level", 
                                         pgp3_log10mean = "Mean Pgp3 IgG level (log10)",
                                         pgp3_log10geomean = "10^pgp3_log10mean",
                                         ct694_mean = "Mean CT694 IgG level", 
                                         ct694_log10mean = "Mean CT694 IgG level (log10)",
                                         ct694_log10geomean = "10^ct694_log10mean", 
                                         pcr_prev = "PCR infection prevalence (%)", 
                                         tf_prev = "Trachomatous Inflammation-Follicular (TF) prevalence (%)", 
                                         ti_prev = "Trachomatous Inflammation-Intense (TI) Prevalence (%)",
                                         pgp3_prev = "Pgp3 IgG seroprevalence (%)", 
                                         ct694_prev = "CT694 IgG seroprevalence (%)",
                                         pgp3ct694_prev = "Pgp3 and CT694 (+ to both) IgG seroprevalence (%)",
                                         
                                         pcr_n_pos_1to5y = "No. of PCR infections detected among 1-5 year olds",
                                         pcr_n_tested_1to5y = "No. of 1-5 year olds tested for infection by PCR",       
                                         pcr_prev_1to5y = "PCR infection prevalence (%) among 1-5 year olds",
                                         tf_n_pos_1to5y = "No. of TF positives among 1-5 year olds",
                                         tf_n_tested_1to5y = "No. of 1-5 year olds tested for TF",
                                         tf_prev_1to5y = "Trachomatous Inflammation-Follicular (TF) prevalence (%) among 1-5 year olds",
                                         ti_n_pos_1to5y = "No. of TI positives among 1-5 year olds",
                                         ti_n_tested_1to5y = "No. of 1-5 year olds tested for TI",
                                         ti_prev_1to5y = "Trachomatous Inflammation-Intense (TI) Prevalence (%) among 1-5 year olds",
                                         pgp3_n_pos_1to5y = "No. of Pgp3 positives among 1-5 year olds",
                                         pgp3_n_tested_1to5y = "No. of 1-5 year olds tested for Pgp3",
                                         pgp3_mean_1to5y = "Mean Pgp3 IgG levels among 1-5 year olds",
                                         pgp3_log10mean_1to5y = "Mean Pgp3 IgG levels (log10) among 1-5 year olds",
                                         pgp3_log10geomean_1to5y = "10^pgp3_n_log10mean_1to5y",
                                         pgp3_prev_1to5y = "Pgp3 IgG seroprevalence (%) among 1-5 year olds",
                                         ct694_n_pos_1to5y = "No. of CT694 seropositives among 1-5 year olds",
                                         ct694_n_tested_1to5y = "No. of 1-5 year olds tested for CT694",
                                         ct694_mean_1to5y = "Mean CT694 IgG levels among 1-5 year olds",
                                         ct694_log10mean_1to5y = "Mean CT694 IgG levels (log10) among 1-5 year olds",
                                         ct694_log10geomean_1to5y = "10^ct694_log10mean_1to5y",
                                         ct694_prev_1to5y = "CT694 IgG seroprevalence (%) among 1-5 year olds",
                                         pgp3ct694_n_pos_1to5y = "No. of Pgp3 and CT694 positives among 1-5 year olds",
                                         pgp3ct694_n_tested_1to5y = "No. of 1-5 year olds tested for Pgp3 and CT694",
                                         pgp3ct694_prev_1to5y = "Pgp3 and CT694 (+ to both) IgG seroprevalence (%) among 1-5 year olds"
                                 )


# labelling factor levels
val_labels(codebook_data_cluster$mda) <- c("Yes" = 1, "No" = 0)

# "If you are not using formr, the codebook package needs to guess which items
# form a scale. The following line finds item aggregates with names like this:
# scale = scale_1 + scale_2R + scale_3R
# identifying these aggregates allows the codebook function to
# automatically compute reliabilities.
# However, it will not reverse items automatically." (R. Arslan)
codebook_data_cluster <- detect_scales(codebook_data_cluster)
```

Create codebook

```
metadata(codebook_data_cluster)$name <- "trachoma_serology_public_data_cluster_v2"
metadata(codebook_data_cluster)$description <- "This is a codebook for the trachoma_serology_public_data_cluster_v2 (cluster level)."

codebook(codebook_data_cluster)
```

### Metadata

#### Description

**Dataset name**: trachoma\_serology\_public\_data\_cluster\_v2

This is a codebook for the trachoma\_serology\_public\_data\_cluster\_v2 (cluster level).

Metadata for search engines

- **Date published**: 2023-04-07

|  |  |  |  |  |  |  |  |  |  |  |  |  |  |  |  |  |  |  |  |  |  |  |  |  |  |  |  |  |  |  |  |  |  |  |  |  |  |  |  |  |  |  |  |  |  |  |  |  |  |  |  |  |  |  |  |  |  |  |
| --- | --- | --- | --- | --- | --- | --- | --- | --- | --- | --- | --- | --- | --- | --- | --- | --- | --- | --- | --- | --- | --- | --- | --- | --- | --- | --- | --- | --- | --- | --- | --- | --- | --- | --- | --- | --- | --- | --- | --- | --- | --- | --- | --- | --- | --- | --- | --- | --- | --- | --- | --- | --- | --- | --- | --- | --- | --- | --- |
| | x | | --- | | study\_id | | cluster\_id\_public | | country | | district | | eu | | eu\_desc | | year | | location\_year\_name | | mda | | pcr\_n\_pos | | pcr\_n\_tested | | tf\_n\_pos | | tf\_n\_tested | | ti\_n\_pos | | ti\_n\_tested | | pgp3\_n\_pos | | pgp3\_n\_tested | | ct694\_n\_pos | | ct694\_n\_tested | | pgp3ct694\_n\_pos | | pgp3ct694\_n\_tested | | pgp3\_mean | | pgp3\_log10mean | | pgp3\_log10geomean | | ct694\_mean | | ct694\_log10mean | | ct694\_log10geomean | | pcr\_prev | | tf\_prev | | ti\_prev | | pgp3\_prev | | ct694\_prev | | pgp3ct694\_prev | | pcr\_n\_pos\_1to5y | | pcr\_n\_tested\_1to5y | | pcr\_prev\_1to5y | | tf\_n\_pos\_1to5y | | tf\_n\_tested\_1to5y | | tf\_prev\_1to5y | | ti\_n\_pos\_1to5y | | ti\_n\_tested\_1to5y | | ti\_prev\_1to5y | | pgp3\_n\_pos\_1to5y | | pgp3\_n\_tested\_1to5y | | pgp3\_mean\_1to5y | | pgp3\_log10mean\_1to5y | | pgp3\_log10geomean\_1to5y | | pgp3\_prev\_1to5y | | ct694\_n\_pos\_1to5y | | ct694\_n\_tested\_1to5y | | ct694\_mean\_1to5y | | ct694\_log10mean\_1to5y | | ct694\_log10geomean\_1to5y | | ct694\_prev\_1to5y | | pgp3ct694\_n\_pos\_1to5y | | pgp3ct694\_n\_tested\_1to5y | | pgp3ct694\_prev\_1to5y | |

#Variables

### study\_id

Study ID

#### Distribution

Distribution of values for study\_id

0 missing values.

#### Summary statistics

| name | label | data\_type | n\_missing | complete\_rate | n\_unique | empty | min | max | whitespace |
| --- | --- | --- | --- | --- | --- | --- | --- | --- | --- |
| study\_id | Study ID | character | 0 | 1 | 9 | 0 | 8 | 16 | 0 |

### cluster\_id\_public

Public cluster ID

#### Distribution

Distribution of values for cluster\_id\_public

0 missing values.

#### Summary statistics

| name | label | data\_type | n\_missing | complete\_rate | n\_unique | empty | min | max | whitespace |
| --- | --- | --- | --- | --- | --- | --- | --- | --- | --- |
| cluster\_id\_public | Public cluster ID | character | 0 | 1 | 482 | 0 | 12 | 27 | 0 |

### country

Country

#### Distribution

Distribution of values for country

0 missing values.

#### Summary statistics

| name | label | data\_type | n\_missing | complete\_rate | n\_unique | empty | min | max | whitespace |
| --- | --- | --- | --- | --- | --- | --- | --- | --- | --- |
| country | Country | character | 0 | 1 | 5 | 0 | 5 | 8 | 0 |

### district

District

#### Distribution

Distribution of values for district

0 missing values.

#### Summary statistics

| name | label | data\_type | n\_missing | complete\_rate | n\_unique | empty | min | max | whitespace |
| --- | --- | --- | --- | --- | --- | --- | --- | --- | --- |
| district | District | character | 0 | 1 | 12 | 0 | 4 | 16 | 0 |

### eu

Evaluation unit

#### Distribution

Distribution of values for eu

0 missing values.

#### Summary statistics

| name | label | data\_type | n\_missing | complete\_rate | n\_unique | empty | min | max | whitespace |
| --- | --- | --- | --- | --- | --- | --- | --- | --- | --- |
| eu | Evaluation unit | character | 0 | 1 | 16 | 0 | 4 | 14 | 0 |

### eu\_desc

Evaluation unit description

#### Distribution

Distribution of values for eu\_desc

0 missing values.

#### Summary statistics

| name | label | data\_type | n\_missing | complete\_rate | n\_unique | empty | min | max | whitespace |
| --- | --- | --- | --- | --- | --- | --- | --- | --- | --- |
| eu\_desc | Evaluation unit description | character | 0 | 1 | 3 | 0 | 3 | 11 | 0 |

### year

Survey year

#### Distribution

Distribution of values for year

0 missing values.

#### Summary statistics

| name | label | data\_type | n\_missing | complete\_rate | n\_unique | empty | min | max | whitespace |
| --- | --- | --- | --- | --- | --- | --- | --- | --- | --- |
| year | Survey year | character | 0 | 1 | 7 | 0 | 4 | 4 | 0 |

### location\_year\_name

Location and year

#### Distribution

Distribution of values for location\_year\_name

0 missing values.

#### Summary statistics

| name | label | data\_type | ordered | value\_labels | n\_missing | complete\_rate | n\_unique | top\_counts |
| --- | --- | --- | --- | --- | --- | --- | --- | --- |
| location\_year\_name | Location and year | factor | FALSE | 1. Agdaz, Morocco 2019, 2. Alefa, Ethiopia 2017, 3. Andabet, Ethiopia 2017, 4. Boumalne Dades, Morocco 2019, 5. Chikwawa, Malawi 2014, 6. Dera, Ethiopia 2017, 7. Dosso, Niger (MORDOR) 2015, 8. Dosso, Niger (MORDOR) 2016, 9. Dosso, Niger (MORDOR) 2017, 10. Dosso, Niger (MORDOR) 2018, 11. Kongwa, Tanzania 2013, 12. Kongwa, Tanzania 2014, 13. Kongwa, Tanzania 2015, 14. Kongwa, Tanzania 2018, 15. Matameye, Niger (PRET) 2013, 16. Mchinji, Malawi 2014, 17. Wag Hemra, Ethiopia (TAITU) 2018, 18. Wag Hemra, Ethiopia (WUHA) 2016, 19. Wag Hemra, Ethiopia (WUHA) 2017, 20. Wag Hemra, Ethiopia (WUHA) 2018, 21. Wag Hemra, Ethiopia (WUHA) 2019, 22. Woreta town, Ethiopia 2017 | 0 | 1 | 22 | Chi: 72, Mch: 72, Kon: 50, Wag: 48 |

### mda

MDA administration within the previous year

#### Distribution

Distribution of values for mda

0 missing values.

#### Summary statistics

| name | label | data\_type | n\_missing | complete\_rate | min | median | max | mean | sd | n\_value\_labels | hist |
| --- | --- | --- | --- | --- | --- | --- | --- | --- | --- | --- | --- |
| mda | MDA administration within the previous year | haven\_labelled | 0 | 1 | 0 | 1 | 1 | 0.6198582 | 0.4857661 | 2 | ▅▁▁▁▁▁▁▇ |

#### Value labels

Response choices

| name | value |
| --- | --- |
| Yes | 1 |
| No | 0 |

### pcr\_n\_pos

No. of PCR infections detected

#### Distribution

Distribution of values for pcr\_n\_pos

0 missing values.

#### Summary statistics

| name | label | data\_type | n\_missing | complete\_rate | min | median | max | mean | sd | hist |
| --- | --- | --- | --- | --- | --- | --- | --- | --- | --- | --- |
| pcr\_n\_pos | No. of PCR infections detected | numeric | 0 | 1 | 0 | 0 | 41 | 2.51773 | 5.660372 | ▇▁▁▁▁ |

### pcr\_n\_tested

No. of individuals tested for infection by PCR

#### Distribution

Distribution of values for pcr\_n\_tested

0 missing values.

#### Summary statistics

| name | label | data\_type | n\_missing | complete\_rate | min | median | max | mean | sd | hist |
| --- | --- | --- | --- | --- | --- | --- | --- | --- | --- | --- |
| pcr\_n\_tested | No. of individuals tested for infection by PCR | numeric | 0 | 1 | 0 | 24 | 313 | 26.03262 | 30.76326 | ▇▁▁▁▁ |

### tf\_n\_pos

No. of individuals positive for TF

#### Distribution

Distribution of values for tf\_n\_pos

0 missing values.

#### Summary statistics

| name | label | data\_type | n\_missing | complete\_rate | min | median | max | mean | sd | hist |
| --- | --- | --- | --- | --- | --- | --- | --- | --- | --- | --- |
| tf\_n\_pos | No. of individuals positive for TF | numeric | 0 | 1 | 0 | 1 | 65 | 7.126241 | 10.57614 | ▇▂▁▁▁ |

### tf\_n\_tested

No. of individuals tested for TF

#### Distribution

Distribution of values for tf\_n\_tested

0 missing values.

#### Summary statistics

| name | label | data\_type | n\_missing | complete\_rate | min | median | max | mean | sd | hist |
| --- | --- | --- | --- | --- | --- | --- | --- | --- | --- | --- |
| tf\_n\_tested | No. of individuals tested for TF | numeric | 0 | 1 | 0 | 34 | 305 | 34.90071 | 37.90357 | ▇▁▁▁▁ |

### ti\_n\_pos

No. of individuals positive for TI

#### Distribution

Distribution of values for ti\_n\_pos

0 missing values.

#### Summary statistics

| name | label | data\_type | n\_missing | complete\_rate | min | median | max | mean | sd | hist |
| --- | --- | --- | --- | --- | --- | --- | --- | --- | --- | --- |
| ti\_n\_pos | No. of individuals positive for TI | numeric | 0 | 1 | 0 | 0 | 18 | 1.817021 | 3.457 | ▇▁▁▁▁ |

### ti\_n\_tested

No. of individuals tested for TI

#### Distribution

Distribution of values for ti\_n\_tested

0 missing values.

#### Summary statistics

| name | label | data\_type | n\_missing | complete\_rate | min | median | max | mean | sd | hist |
| --- | --- | --- | --- | --- | --- | --- | --- | --- | --- | --- |
| ti\_n\_tested | No. of individuals tested for TI | numeric | 0 | 1 | 0 | 20 | 305 | 28.20142 | 38.17029 | ▇▁▁▁▁ |

### pgp3\_n\_pos

No. of positive Pgp3 response

#### Distribution

Distribution of values for pgp3\_n\_pos

0 missing values.

#### Summary statistics

| name | label | data\_type | n\_missing | complete\_rate | min | median | max | mean | sd | hist |
| --- | --- | --- | --- | --- | --- | --- | --- | --- | --- | --- |
| pgp3\_n\_pos | No. of positive Pgp3 response | numeric | 0 | 1 | 0 | 4 | 137 | 9.021277 | 14.81719 | ▇▁▁▁▁ |

### pgp3\_n\_tested

No. of individuals tested for Pgp3

#### Distribution

Distribution of values for pgp3\_n\_tested

0 missing values.

#### Summary statistics

| name | label | data\_type | n\_missing | complete\_rate | min | median | max | mean | sd | hist |
| --- | --- | --- | --- | --- | --- | --- | --- | --- | --- | --- |
| pgp3\_n\_tested | No. of individuals tested for Pgp3 | numeric | 0 | 1 | 1 | 38 | 305 | 43.47092 | 31.74395 | ▇▁▁▁▁ |

### ct694\_n\_pos

No. of positive CT694 response

#### Distribution

Distribution of values for ct694\_n\_pos

0 missing values.

#### Summary statistics

| name | label | data\_type | n\_missing | complete\_rate | min | median | max | mean | sd | hist |
| --- | --- | --- | --- | --- | --- | --- | --- | --- | --- | --- |
| ct694\_n\_pos | No. of positive CT694 response | numeric | 0 | 1 | 0 | 1 | 145 | 7.011348 | 14.62922 | ▇▁▁▁▁ |

### ct694\_n\_tested

No. of individuals tested for CT694

#### Distribution

Distribution of values for ct694\_n\_tested

0 missing values.

#### Summary statistics

| name | label | data\_type | n\_missing | complete\_rate | min | median | max | mean | sd | hist |
| --- | --- | --- | --- | --- | --- | --- | --- | --- | --- | --- |
| ct694\_n\_tested | No. of individuals tested for CT694 | numeric | 0 | 1 | 0 | 28 | 305 | 31.69787 | 36.87095 | ▇▁▁▁▁ |

### pgp3ct694\_n\_pos

No. Pgp3 and CT694 seropositives

#### Distribution

Distribution of values for pgp3ct694\_n\_pos

0 missing values.

#### Summary statistics

| name | label | data\_type | n\_missing | complete\_rate | min | median | max | mean | sd | hist |
| --- | --- | --- | --- | --- | --- | --- | --- | --- | --- | --- |
| pgp3ct694\_n\_pos | No. Pgp3 and CT694 seropositives | numeric | 0 | 1 | 0 | 0 | 133 | 6.380142 | 13.67428 | ▇▁▁▁▁ |

### pgp3ct694\_n\_tested

No. of individuals tested for Pgp3 and CT694

#### Distribution

Distribution of values for pgp3ct694\_n\_tested

0 missing values.

#### Summary statistics

| name | label | data\_type | n\_missing | complete\_rate | min | median | max | mean | sd | hist |
| --- | --- | --- | --- | --- | --- | --- | --- | --- | --- | --- |
| pgp3ct694\_n\_tested | No. of individuals tested for Pgp3 and CT694 | numeric | 0 | 1 | 0 | 28 | 305 | 31.69787 | 36.87095 | ▇▁▁▁▁ |

### pgp3\_mean

Mean Pgp3 IgG level

#### Distribution

Distribution of values for pgp3\_mean

144 missing values.

#### Summary statistics

| name | label | data\_type | n\_missing | complete\_rate | min | median | max | mean | sd | hist |
| --- | --- | --- | --- | --- | --- | --- | --- | --- | --- | --- |
| pgp3\_mean | Mean Pgp3 IgG level | numeric | 144 | 0.7957447 | -3 | 2029 | 24866 | 4778.12 | 5766.849 | ▇▂▂▁▁ |

### pgp3\_log10mean

Mean Pgp3 IgG level (log10)

#### Distribution

Distribution of values for pgp3\_log10mean

144 missing values.

#### Summary statistics

| name | label | data\_type | n\_missing | complete\_rate | min | median | max | mean | sd | hist |
| --- | --- | --- | --- | --- | --- | --- | --- | --- | --- | --- |
| pgp3\_log10mean | Mean Pgp3 IgG level (log10) | numeric | 144 | 0.7957447 | 0 | 1.2 | 4 | 1.555859 | 0.910642 | ▆▇▅▃▁ |

### pgp3\_log10geomean

10^pgp3\_log10mean

#### Distribution

Distribution of values for pgp3\_log10geomean

144 missing values.

#### Summary statistics

| name | label | data\_type | n\_missing | complete\_rate | min | median | max | mean | sd | hist |
| --- | --- | --- | --- | --- | --- | --- | --- | --- | --- | --- |
| pgp3\_log10geomean | 10^pgp3\_log10mean | numeric | 144 | 0.7957447 | 1 | 17 | 10178 | 338.4304 | 1012.477 | ▇▁▁▁▁ |

### ct694\_mean

Mean CT694 IgG level

#### Distribution

Distribution of values for ct694\_mean

194 missing values.

#### Summary statistics

| name | label | data\_type | n\_missing | complete\_rate | min | median | max | mean | sd | hist |
| --- | --- | --- | --- | --- | --- | --- | --- | --- | --- | --- |
| ct694\_mean | Mean CT694 IgG level | numeric | 194 | 0.7248227 | 1.5 | 549 | 10556 | 1827.647 | 2382.564 | ▇▂▁▁▁ |

### ct694\_log10mean

Mean CT694 IgG level (log10)

#### Distribution

Distribution of values for ct694\_log10mean

194 missing values.

#### Summary statistics

| name | label | data\_type | n\_missing | complete\_rate | min | median | max | mean | sd | hist |
| --- | --- | --- | --- | --- | --- | --- | --- | --- | --- | --- |
| ct694\_log10mean | Mean CT694 IgG level (log10) | numeric | 194 | 0.7248227 | 0.15 | 1.5 | 3.5 | 1.592659 | 0.6886789 | ▃▇▅▅▁ |

### ct694\_log10geomean

10^ct694\_log10mean

#### Distribution

Distribution of values for ct694\_log10geomean

194 missing values.

#### Summary statistics

| name | label | data\_type | n\_missing | complete\_rate | min | median | max | mean | sd | hist |
| --- | --- | --- | --- | --- | --- | --- | --- | --- | --- | --- |
| ct694\_log10geomean | 10^ct694\_log10mean | numeric | 194 | 0.7248227 | 1.4 | 29 | 2901 | 140.246 | 306.2819 | ▇▁▁▁▁ |

### pcr\_prev

PCR infection prevalence (%)

#### Distribution

Distribution of values for pcr\_prev

286 missing values.

#### Summary statistics

| name | label | data\_type | n\_missing | complete\_rate | min | median | max | mean | sd | hist |
| --- | --- | --- | --- | --- | --- | --- | --- | --- | --- | --- |
| pcr\_prev | PCR infection prevalence (%) | numeric | 286 | 0.5943262 | 0 | 2.4 | 73 | 11.11555 | 16.16703 | ▇▂▁▁▁ |

### tf\_prev

Trachomatous Inflammation-Follicular (TF) prevalence (%)

#### Distribution

Distribution of values for tf\_prev

119 missing values.

#### Summary statistics

| name | label | data\_type | n\_missing | complete\_rate | min | median | max | mean | sd | hist |
| --- | --- | --- | --- | --- | --- | --- | --- | --- | --- | --- |
| tf\_prev | Trachomatous Inflammation-Follicular (TF) prevalence (%) | numeric | 119 | 0.8312057 | 0 | 11 | 93 | 24.01752 | 26.33594 | ▇▁▂▂▁ |

### ti\_prev

Trachomatous Inflammation-Intense (TI) Prevalence (%)

#### Distribution

Distribution of values for ti\_prev

169 missing values.

#### Summary statistics

| name | label | data\_type | n\_missing | complete\_rate | min | median | max | mean | sd | hist |
| --- | --- | --- | --- | --- | --- | --- | --- | --- | --- | --- |
| ti\_prev | Trachomatous Inflammation-Intense (TI) Prevalence (%) | numeric | 169 | 0.7602837 | 0 | 0 | 90 | 9.909822 | 16.49561 | ▇▂▁▁▁ |

### pgp3\_prev

Pgp3 IgG seroprevalence (%)

#### Distribution

Distribution of values for pgp3\_prev

0 missing values.

#### Summary statistics

| name | label | data\_type | n\_missing | complete\_rate | min | median | max | mean | sd | hist |
| --- | --- | --- | --- | --- | --- | --- | --- | --- | --- | --- |
| pgp3\_prev | Pgp3 IgG seroprevalence (%) | numeric | 0 | 1 | 0 | 9.7 | 88 | 19.16727 | 21.87402 | ▇▂▂▁▁ |

### ct694\_prev

CT694 IgG seroprevalence (%)

#### Distribution

Distribution of values for ct694\_prev

194 missing values.

#### Summary statistics

| name | label | data\_type | n\_missing | complete\_rate | min | median | max | mean | sd | hist |
| --- | --- | --- | --- | --- | --- | --- | --- | --- | --- | --- |
| ct694\_prev | CT694 IgG seroprevalence (%) | numeric | 194 | 0.7248227 | 0 | 10 | 83 | 19.78319 | 21.07098 | ▇▂▂▁▁ |

### pgp3ct694\_prev

Pgp3 and CT694 (+ to both) IgG seroprevalence (%)

#### Distribution

Distribution of values for pgp3ct694\_prev

194 missing values.

#### Summary statistics

| name | label | data\_type | n\_missing | complete\_rate | min | median | max | mean | sd | hist |
| --- | --- | --- | --- | --- | --- | --- | --- | --- | --- | --- |
| pgp3ct694\_prev | Pgp3 and CT694 (+ to both) IgG seroprevalence (%) | numeric | 194 | 0.7248227 | 0 | 8 | 81 | 18.1183 | 21.20252 | ▇▂▂▁▁ |

### pcr\_n\_pos\_1to5y

No. of PCR infections detected among 1-5 year olds

#### Distribution

Distribution of values for pcr\_n\_pos\_1to5y

1 missing values.

#### Summary statistics

| name | label | data\_type | n\_missing | complete\_rate | min | median | max | mean | sd | hist |
| --- | --- | --- | --- | --- | --- | --- | --- | --- | --- | --- |
| pcr\_n\_pos\_1to5y | No. of PCR infections detected among 1-5 year olds | numeric | 1 | 0.9985816 | 0 | 0 | 23 | 1.940341 | 4.054658 | ▇▁▁▁▁ |

### pcr\_n\_tested\_1to5y

No. of 1-5 year olds tested for infection by PCR

#### Distribution

Distribution of values for pcr\_n\_tested\_1to5y

1 missing values.

#### Summary statistics

| name | label | data\_type | n\_missing | complete\_rate | min | median | max | mean | sd | hist |
| --- | --- | --- | --- | --- | --- | --- | --- | --- | --- | --- |
| pcr\_n\_tested\_1to5y | No. of 1-5 year olds tested for infection by PCR | numeric | 1 | 0.9985816 | 0 | 20 | 115 | 16.98864 | 18.71186 | ▇▅▁▁▁ |

### pcr\_prev\_1to5y

PCR infection prevalence (%) among 1-5 year olds

#### Distribution

Distribution of values for pcr\_prev\_1to5y

293 missing values.

#### Summary statistics

| name | label | data\_type | n\_missing | complete\_rate | min | median | max | mean | sd | hist |
| --- | --- | --- | --- | --- | --- | --- | --- | --- | --- | --- |
| pcr\_prev\_1to5y | PCR infection prevalence (%) among 1-5 year olds | numeric | 293 | 0.5843972 | 0 | 3.1 | 73 | 11.87218 | 17.09074 | ▇▂▁▁▁ |

### tf\_n\_pos\_1to5y

No. of TF positives among 1-5 year olds

#### Distribution

Distribution of values for tf\_n\_pos\_1to5y

1 missing values.

#### Summary statistics

| name | label | data\_type | n\_missing | complete\_rate | min | median | max | mean | sd | hist |
| --- | --- | --- | --- | --- | --- | --- | --- | --- | --- | --- |
| tf\_n\_pos\_1to5y | No. of TF positives among 1-5 year olds | numeric | 1 | 0.9985816 | 0 | 1 | 60 | 5.620739 | 8.073607 | ▇▂▁▁▁ |

### tf\_n\_tested\_1to5y

No. of 1-5 year olds tested for TF

#### Distribution

Distribution of values for tf\_n\_tested\_1to5y

3 missing values.

#### Summary statistics

| name | label | data\_type | n\_missing | complete\_rate | min | median | max | mean | sd | hist |
| --- | --- | --- | --- | --- | --- | --- | --- | --- | --- | --- |
| tf\_n\_tested\_1to5y | No. of 1-5 year olds tested for TF | numeric | 3 | 0.9957447 | 0 | 22 | 170 | 22.5114 | 23.88845 | ▇▁▁▁▁ |

### tf\_prev\_1to5y

Trachomatous Inflammation-Follicular (TF) prevalence (%) among 1-5 year olds

#### Distribution

Distribution of values for tf\_prev\_1to5y

198 missing values.

#### Summary statistics

| name | label | data\_type | n\_missing | complete\_rate | min | median | max | mean | sd | hist |
| --- | --- | --- | --- | --- | --- | --- | --- | --- | --- | --- |
| tf\_prev\_1to5y | Trachomatous Inflammation-Follicular (TF) prevalence (%) among 1-5 year olds | numeric | 198 | 0.7191489 | 0 | 13 | 100 | 27.27863 | 28.31703 | ▇▁▂▂▁ |

### ti\_n\_pos\_1to5y

No. of TI positives among 1-5 year olds

#### Distribution

Distribution of values for ti\_n\_pos\_1to5y

1 missing values.

#### Summary statistics

| name | label | data\_type | n\_missing | complete\_rate | min | median | max | mean | sd | hist |
| --- | --- | --- | --- | --- | --- | --- | --- | --- | --- | --- |
| ti\_n\_pos\_1to5y | No. of TI positives among 1-5 year olds | numeric | 1 | 0.9985816 | 0 | 0 | 16 | 1.539773 | 2.922149 | ▇▁▁▁▁ |

### ti\_n\_tested\_1to5y

No. of 1-5 year olds tested for TI

#### Distribution

Distribution of values for ti\_n\_tested\_1to5y

1 missing values.

#### Summary statistics

| name | label | data\_type | n\_missing | complete\_rate | min | median | max | mean | sd | hist |
| --- | --- | --- | --- | --- | --- | --- | --- | --- | --- | --- |
| ti\_n\_tested\_1to5y | No. of 1-5 year olds tested for TI | numeric | 1 | 0.9985816 | 0 | 15 | 170 | 18.47727 | 24.75863 | ▇▁▁▁▁ |

### ti\_prev\_1to5y

Trachomatous Inflammation-Intense (TI) Prevalence (%) among 1-5 year olds

#### Distribution

Distribution of values for ti\_prev\_1to5y

248 missing values.

#### Summary statistics

| name | label | data\_type | n\_missing | complete\_rate | min | median | max | mean | sd | hist |
| --- | --- | --- | --- | --- | --- | --- | --- | --- | --- | --- |
| ti\_prev\_1to5y | Trachomatous Inflammation-Intense (TI) Prevalence (%) among 1-5 year olds | numeric | 248 | 0.648227 | 0 | 0 | 100 | 12.57608 | 18.8375 | ▇▂▁▁▁ |

### pgp3\_n\_pos\_1to5y

No. of Pgp3 positives among 1-5 year olds

#### Distribution

Distribution of values for pgp3\_n\_pos\_1to5y

1 missing values.

#### Summary statistics

| name | label | data\_type | n\_missing | complete\_rate | min | median | max | mean | sd | hist |
| --- | --- | --- | --- | --- | --- | --- | --- | --- | --- | --- |
| pgp3\_n\_pos\_1to5y | No. of Pgp3 positives among 1-5 year olds | numeric | 1 | 0.9985816 | 0 | 1 | 54 | 4.832386 | 7.398456 | ▇▂▁▁▁ |

### pgp3\_n\_tested\_1to5y

No. of 1-5 year olds tested for Pgp3

#### Distribution

Distribution of values for pgp3\_n\_tested\_1to5y

1 missing values.

#### Summary statistics

| name | label | data\_type | n\_missing | complete\_rate | min | median | max | mean | sd | hist |
| --- | --- | --- | --- | --- | --- | --- | --- | --- | --- | --- |
| pgp3\_n\_tested\_1to5y | No. of 1-5 year olds tested for Pgp3 | numeric | 1 | 0.9985816 | 1 | 27 | 170 | 29.75568 | 19.4155 | ▇▂▁▁▁ |

### pgp3\_mean\_1to5y

Mean Pgp3 IgG levels among 1-5 year olds

#### Distribution

Distribution of values for pgp3\_mean\_1to5y

145 missing values.

#### Summary statistics

| name | label | data\_type | n\_missing | complete\_rate | min | median | max | mean | sd | hist |
| --- | --- | --- | --- | --- | --- | --- | --- | --- | --- | --- |
| pgp3\_mean\_1to5y | Mean Pgp3 IgG levels among 1-5 year olds | numeric | 145 | 0.7943262 | 0 | 1341 | 24866 | 4352.261 | 5658.441 | ▇▂▂▁▁ |

### pgp3\_log10mean\_1to5y

Mean Pgp3 IgG levels (log10) among 1-5 year olds

#### Distribution

Distribution of values for pgp3\_log10mean\_1to5y

145 missing values.

#### Summary statistics

| name | label | data\_type | n\_missing | complete\_rate | min | median | max | mean | sd | hist |
| --- | --- | --- | --- | --- | --- | --- | --- | --- | --- | --- |
| pgp3\_log10mean\_1to5y | Mean Pgp3 IgG levels (log10) among 1-5 year olds | numeric | 145 | 0.7943262 | 0 | 1.1 | 4.1 | 1.442767 | 0.8749414 | ▇▇▅▃▁ |

### pgp3\_log10geomean\_1to5y

10^pgp3\_n\_log10mean\_1to5y

#### Distribution

Distribution of values for pgp3\_log10geomean\_1to5y

145 missing values.

#### Summary statistics

| name | label | data\_type | n\_missing | complete\_rate | min | median | max | mean | sd | hist |
| --- | --- | --- | --- | --- | --- | --- | --- | --- | --- | --- |
| pgp3\_log10geomean\_1to5y | 10^pgp3\_n\_log10mean\_1to5y | numeric | 145 | 0.7943262 | 1 | 12 | 12404 | 273.9217 | 960.4725 | ▇▁▁▁▁ |

### pgp3\_prev\_1to5y

Pgp3 IgG seroprevalence (%) among 1-5 year olds

#### Distribution

Distribution of values for pgp3\_prev\_1to5y

1 missing values.

#### Summary statistics

| name | label | data\_type | n\_missing | complete\_rate | min | median | max | mean | sd | hist |
| --- | --- | --- | --- | --- | --- | --- | --- | --- | --- | --- |
| pgp3\_prev\_1to5y | Pgp3 IgG seroprevalence (%) among 1-5 year olds | numeric | 1 | 0.9985816 | 0 | 5.7 | 93 | 16.1309 | 20.6599 | ▇▂▂▁▁ |

### ct694\_n\_pos\_1to5y

No. of CT694 seropositives among 1-5 year olds

#### Distribution

Distribution of values for ct694\_n\_pos\_1to5y

1 missing values.

#### Summary statistics

| name | label | data\_type | n\_missing | complete\_rate | min | median | max | mean | sd | hist |
| --- | --- | --- | --- | --- | --- | --- | --- | --- | --- | --- |
| ct694\_n\_pos\_1to5y | No. of CT694 seropositives among 1-5 year olds | numeric | 1 | 0.9985816 | 0 | 1 | 61 | 4.015625 | 7.105267 | ▇▁▁▁▁ |

### ct694\_n\_tested\_1to5y

No. of 1-5 year olds tested for CT694

#### Distribution

Distribution of values for ct694\_n\_tested\_1to5y

1 missing values.

#### Summary statistics

| name | label | data\_type | n\_missing | complete\_rate | min | median | max | mean | sd | hist |
| --- | --- | --- | --- | --- | --- | --- | --- | --- | --- | --- |
| ct694\_n\_tested\_1to5y | No. of 1-5 year olds tested for CT694 | numeric | 1 | 0.9985816 | 0 | 21 | 170 | 22.61932 | 23.51834 | ▇▂▁▁▁ |

### ct694\_mean\_1to5y

Mean CT694 IgG levels among 1-5 year olds

#### Distribution

Distribution of values for ct694\_mean\_1to5y

195 missing values.

#### Summary statistics

| name | label | data\_type | n\_missing | complete\_rate | min | median | max | mean | sd | hist |
| --- | --- | --- | --- | --- | --- | --- | --- | --- | --- | --- |
| ct694\_mean\_1to5y | Mean CT694 IgG levels among 1-5 year olds | numeric | 195 | 0.7234043 | 1.5 | 340 | 11022 | 1738.893 | 2379.704 | ▇▂▁▁▁ |

### ct694\_log10mean\_1to5y

Mean CT694 IgG levels (log10) among 1-5 year olds

#### Distribution

Distribution of values for ct694\_log10mean\_1to5y

195 missing values.

#### Summary statistics

| name | label | data\_type | n\_missing | complete\_rate | min | median | max | mean | sd | hist |
| --- | --- | --- | --- | --- | --- | --- | --- | --- | --- | --- |
| ct694\_log10mean\_1to5y | Mean CT694 IgG levels (log10) among 1-5 year olds | numeric | 195 | 0.7234043 | 0.15 | 1.4 | 3.5 | 1.554268 | 0.6577118 | ▂▇▅▃▁ |

### ct694\_log10geomean\_1to5y

10^ct694\_log10mean\_1to5y

#### Distribution

Distribution of values for ct694\_log10geomean\_1to5y

195 missing values.

#### Summary statistics

| name | label | data\_type | n\_missing | complete\_rate | min | median | max | mean | sd | hist |
| --- | --- | --- | --- | --- | --- | --- | --- | --- | --- | --- |
| ct694\_log10geomean\_1to5y | 10^ct694\_log10mean\_1to5y | numeric | 195 | 0.7234043 | 1.4 | 27 | 2901 | 124.7837 | 294.2881 | ▇▁▁▁▁ |

### ct694\_prev\_1to5y

CT694 IgG seroprevalence (%) among 1-5 year olds

#### Distribution

Distribution of values for ct694\_prev\_1to5y

195 missing values.

#### Summary statistics

| name | label | data\_type | n\_missing | complete\_rate | min | median | max | mean | sd | hist |
| --- | --- | --- | --- | --- | --- | --- | --- | --- | --- | --- |
| ct694\_prev\_1to5y | CT694 IgG seroprevalence (%) among 1-5 year olds | numeric | 195 | 0.7234043 | 0 | 8.7 | 83 | 17.65809 | 19.85623 | ▇▃▂▁▁ |

### pgp3ct694\_n\_pos\_1to5y

No. of Pgp3 and CT694 positives among 1-5 year olds

#### Distribution

Distribution of values for pgp3ct694\_n\_pos\_1to5y

1 missing values.

#### Summary statistics

| name | label | data\_type | n\_missing | complete\_rate | min | median | max | mean | sd | hist |
| --- | --- | --- | --- | --- | --- | --- | --- | --- | --- | --- |
| pgp3ct694\_n\_pos\_1to5y | No. of Pgp3 and CT694 positives among 1-5 year olds | numeric | 1 | 0.9985816 | 0 | 0 | 51 | 3.559659 | 6.621175 | ▇▁▁▁▁ |

### pgp3ct694\_n\_tested\_1to5y

No. of 1-5 year olds tested for Pgp3 and CT694

#### Distribution

Distribution of values for pgp3ct694\_n\_tested\_1to5y

1 missing values.

#### Summary statistics

| name | label | data\_type | n\_missing | complete\_rate | min | median | max | mean | sd | hist |
| --- | --- | --- | --- | --- | --- | --- | --- | --- | --- | --- |
| pgp3ct694\_n\_tested\_1to5y | No. of 1-5 year olds tested for Pgp3 and CT694 | numeric | 1 | 0.9985816 | 0 | 21 | 170 | 22.61932 | 23.51834 | ▇▂▁▁▁ |

### pgp3ct694\_prev\_1to5y

Pgp3 and CT694 (+ to both) IgG seroprevalence (%) among 1-5 year olds

#### Distribution

Distribution of values for pgp3ct694\_prev\_1to5y

195 missing values.

#### Summary statistics

| name | label | data\_type | n\_missing | complete\_rate | min | median | max | mean | sd | hist |
| --- | --- | --- | --- | --- | --- | --- | --- | --- | --- | --- |
| pgp3ct694\_prev\_1to5y | Pgp3 and CT694 (+ to both) IgG seroprevalence (%) among 1-5 year olds | numeric | 195 | 0.7234043 | 0 | 5.7 | 80 | 16.04093 | 20.0139 | ▇▂▂▁▁ |

## Missingness report

## Codebook table

| name | label | data\_type | ordered | value\_labels | n\_missing | complete\_rate | n\_unique | empty | top\_counts | min | median | max | mean | sd | whitespace | n\_value\_labels | hist |
| --- | --- | --- | --- | --- | --- | --- | --- | --- | --- | --- | --- | --- | --- | --- | --- | --- | --- |
| study\_id | Study ID | character | NA | NA | 0 | 1.0000000 | 9 | 0 | NA | 8 | NA | 16 | NA | NA | 0 | NA | NA |
| cluster\_id\_public | Public cluster ID | character | NA | NA | 0 | 1.0000000 | 482 | 0 | NA | 12 | NA | 27 | NA | NA | 0 | NA | NA |
| country | Country | character | NA | NA | 0 | 1.0000000 | 5 | 0 | NA | 5 | NA | 8 | NA | NA | 0 | NA | NA |
| district | District | character | NA | NA | 0 | 1.0000000 | 12 | 0 | NA | 4 | NA | 16 | NA | NA | 0 | NA | NA |
| eu | Evaluation unit | character | NA | NA | 0 | 1.0000000 | 16 | 0 | NA | 4 | NA | 14 | NA | NA | 0 | NA | NA |
| eu\_desc | Evaluation unit description | character | NA | NA | 0 | 1.0000000 | 3 | 0 | NA | 3 | NA | 11 | NA | NA | 0 | NA | NA |
| year | Survey year | character | NA | NA | 0 | 1.0000000 | 7 | 0 | NA | 4 | NA | 4 | NA | NA | 0 | NA | NA |
| location\_year\_name | Location and year | factor | FALSE | 1. Agdaz, Morocco 2019, 2. Alefa, Ethiopia 2017, 3. Andabet, Ethiopia 2017, 4. Boumalne Dades, Morocco 2019, 5. Chikwawa, Malawi 2014, 6. Dera, Ethiopia 2017, 7. Dosso, Niger (MORDOR) 2015, 8. Dosso, Niger (MORDOR) 2016, 9. Dosso, Niger (MORDOR) 2017, 10. Dosso, Niger (MORDOR) 2018, 11. Kongwa, Tanzania 2013, 12. Kongwa, Tanzania 2014, 13. Kongwa, Tanzania 2015, 14. Kongwa, Tanzania 2018, 15. Matameye, Niger (PRET) 2013, 16. Mchinji, Malawi 2014, 17. Wag Hemra, Ethiopia (TAITU) 2018, 18. Wag Hemra, Ethiopia (WUHA) 2016, 19. Wag Hemra, Ethiopia (WUHA) 2017, 20. Wag Hemra, Ethiopia (WUHA) 2018, 21. Wag Hemra, Ethiopia (WUHA) 2019, 22. Woreta town, Ethiopia 2017 | 0 | 1.0000000 | 22 | NA | Chi: 72, Mch: 72, Kon: 50, Wag: 48 | NA | NA | NA | NA | NA | NA | NA | NA |
| mda | MDA administration within the previous year | haven\_labelled | NA | 1. Yes, 0. No | 0 | 1.0000000 | NA | NA | NA | 0 | 1 | 1 | 0.6198582 | 0.4857661 | NA | 2 | ▅▁▁▁▁▁▁▇ |
| pcr\_n\_pos | No. of PCR infections detected | numeric | NA | NA | 0 | 1.0000000 | NA | NA | NA | 0.00 | 0.0 | 41.0 | 2.5177305 | 5.6603722 | NA | NA | ▇▁▁▁▁ |
| pcr\_n\_tested | No. of individuals tested for infection by PCR | numeric | NA | NA | 0 | 1.0000000 | NA | NA | NA | 0.00 | 24.0 | 313.0 | 26.0326241 | 30.7632605 | NA | NA | ▇▁▁▁▁ |
| tf\_n\_pos | No. of individuals positive for TF | numeric | NA | NA | 0 | 1.0000000 | NA | NA | NA | 0.00 | 1.0 | 65.0 | 7.1262411 | 10.5761420 | NA | NA | ▇▂▁▁▁ |
| tf\_n\_tested | No. of individuals tested for TF | numeric | NA | NA | 0 | 1.0000000 | NA | NA | NA | 0.00 | 34.0 | 305.0 | 34.9007092 | 37.9035680 | NA | NA | ▇▁▁▁▁ |
| ti\_n\_pos | No. of individuals positive for TI | numeric | NA | NA | 0 | 1.0000000 | NA | NA | NA | 0.00 | 0.0 | 18.0 | 1.8170213 | 3.4569996 | NA | NA | ▇▁▁▁▁ |
| ti\_n\_tested | No. of individuals tested for TI | numeric | NA | NA | 0 | 1.0000000 | NA | NA | NA | 0.00 | 20.0 | 305.0 | 28.2014184 | 38.1702860 | NA | NA | ▇▁▁▁▁ |
| pgp3\_n\_pos | No. of positive Pgp3 response | numeric | NA | NA | 0 | 1.0000000 | NA | NA | NA | 0.00 | 4.0 | 137.0 | 9.0212766 | 14.8171948 | NA | NA | ▇▁▁▁▁ |
| pgp3\_n\_tested | No. of individuals tested for Pgp3 | numeric | NA | NA | 0 | 1.0000000 | NA | NA | NA | 1.00 | 38.0 | 305.0 | 43.4709220 | 31.7439520 | NA | NA | ▇▁▁▁▁ |
| ct694\_n\_pos | No. of positive CT694 response | numeric | NA | NA | 0 | 1.0000000 | NA | NA | NA | 0.00 | 1.0 | 145.0 | 7.0113475 | 14.6292199 | NA | NA | ▇▁▁▁▁ |
| ct694\_n\_tested | No. of individuals tested for CT694 | numeric | NA | NA | 0 | 1.0000000 | NA | NA | NA | 0.00 | 28.0 | 305.0 | 31.6978723 | 36.8709483 | NA | NA | ▇▁▁▁▁ |
| pgp3ct694\_n\_pos | No. Pgp3 and CT694 seropositives | numeric | NA | NA | 0 | 1.0000000 | NA | NA | NA | 0.00 | 0.0 | 133.0 | 6.3801418 | 13.6742813 | NA | NA | ▇▁▁▁▁ |
| pgp3ct694\_n\_tested | No. of individuals tested for Pgp3 and CT694 | numeric | NA | NA | 0 | 1.0000000 | NA | NA | NA | 0.00 | 28.0 | 305.0 | 31.6978723 | 36.8709483 | NA | NA | ▇▁▁▁▁ |
| pgp3\_mean | Mean Pgp3 IgG level | numeric | NA | NA | 144 | 0.7957447 | NA | NA | NA | -3.00 | 2028.8 | 24866.3 | 4778.1198327 | 5766.8492973 | NA | NA | ▇▂▂▁▁ |
| pgp3\_log10mean | Mean Pgp3 IgG level (log10) | numeric | NA | NA | 144 | 0.7957447 | NA | NA | NA | 0.00 | 1.2 | 4.0 | 1.5558590 | 0.9106420 | NA | NA | ▆▇▅▃▁ |
| pgp3\_log10geomean | 10^pgp3\_log10mean | numeric | NA | NA | 144 | 0.7957447 | NA | NA | NA | 1.00 | 17.2 | 10178.1 | 338.4303652 | 1012.4770571 | NA | NA | ▇▁▁▁▁ |
| ct694\_mean | Mean CT694 IgG level | numeric | NA | NA | 194 | 0.7248227 | NA | NA | NA | 1.50 | 548.7 | 10555.5 | 1827.6472709 | 2382.5642633 | NA | NA | ▇▂▁▁▁ |
| ct694\_log10mean | Mean CT694 IgG level (log10) | numeric | NA | NA | 194 | 0.7248227 | NA | NA | NA | 0.15 | 1.5 | 3.5 | 1.5926594 | 0.6886789 | NA | NA | ▃▇▅▅▁ |
| ct694\_log10geomean | 10^ct694\_log10mean | numeric | NA | NA | 194 | 0.7248227 | NA | NA | NA | 1.41 | 28.6 | 2900.6 | 140.2459569 | 306.2818651 | NA | NA | ▇▁▁▁▁ |
| pcr\_prev | PCR infection prevalence (%) | numeric | NA | NA | 286 | 0.5943262 | NA | NA | NA | 0.00 | 2.4 | 72.7 | 11.1155514 | 16.1670256 | NA | NA | ▇▂▁▁▁ |
| tf\_prev | Trachomatous Inflammation-Follicular (TF) prevalence (%) | numeric | NA | NA | 119 | 0.8312057 | NA | NA | NA | 0.00 | 10.5 | 93.1 | 24.0175243 | 26.3359443 | NA | NA | ▇▁▂▂▁ |
| ti\_prev | Trachomatous Inflammation-Intense (TI) Prevalence (%) | numeric | NA | NA | 169 | 0.7602837 | NA | NA | NA | 0.00 | 0.0 | 90.0 | 9.9098222 | 16.4956105 | NA | NA | ▇▂▁▁▁ |
| pgp3\_prev | Pgp3 IgG seroprevalence (%) | numeric | NA | NA | 0 | 1.0000000 | NA | NA | NA | 0.00 | 9.7 | 87.5 | 19.1672654 | 21.8740207 | NA | NA | ▇▂▂▁▁ |
| ct694\_prev | CT694 IgG seroprevalence (%) | numeric | NA | NA | 194 | 0.7248227 | NA | NA | NA | 0.00 | 10.0 | 83.3 | 19.7831901 | 21.0709802 | NA | NA | ▇▂▂▁▁ |
| pgp3ct694\_prev | Pgp3 and CT694 (+ to both) IgG seroprevalence (%) | numeric | NA | NA | 194 | 0.7248227 | NA | NA | NA | 0.00 | 8.0 | 80.9 | 18.1182979 | 21.2025179 | NA | NA | ▇▂▂▁▁ |
| pcr\_n\_pos\_1to5y | No. of PCR infections detected among 1-5 year olds | numeric | NA | NA | 1 | 0.9985816 | NA | NA | NA | 0.00 | 0.0 | 23.0 | 1.9403409 | 4.0546576 | NA | NA | ▇▁▁▁▁ |
| pcr\_n\_tested\_1to5y | No. of 1-5 year olds tested for infection by PCR | numeric | NA | NA | 1 | 0.9985816 | NA | NA | NA | 0.00 | 20.0 | 115.0 | 16.9886364 | 18.7118568 | NA | NA | ▇▅▁▁▁ |
| pcr\_prev\_1to5y | PCR infection prevalence (%) among 1-5 year olds | numeric | NA | NA | 293 | 0.5843972 | NA | NA | NA | 0.00 | 3.1 | 72.7 | 11.8721794 | 17.0907384 | NA | NA | ▇▂▁▁▁ |
| tf\_n\_pos\_1to5y | No. of TF positives among 1-5 year olds | numeric | NA | NA | 1 | 0.9985816 | NA | NA | NA | 0.00 | 1.0 | 60.0 | 5.6207386 | 8.0736067 | NA | NA | ▇▂▁▁▁ |
| tf\_n\_tested\_1to5y | No. of 1-5 year olds tested for TF | numeric | NA | NA | 3 | 0.9957447 | NA | NA | NA | 0.00 | 22.0 | 170.0 | 22.5113960 | 23.8884536 | NA | NA | ▇▁▁▁▁ |
| tf\_prev\_1to5y | Trachomatous Inflammation-Follicular (TF) prevalence (%) among 1-5 year olds | numeric | NA | NA | 198 | 0.7191489 | NA | NA | NA | 0.00 | 13.5 | 100.0 | 27.2786325 | 28.3170318 | NA | NA | ▇▁▂▂▁ |
| ti\_n\_pos\_1to5y | No. of TI positives among 1-5 year olds | numeric | NA | NA | 1 | 0.9985816 | NA | NA | NA | 0.00 | 0.0 | 16.0 | 1.5397727 | 2.9221493 | NA | NA | ▇▁▁▁▁ |
| ti\_n\_tested\_1to5y | No. of 1-5 year olds tested for TI | numeric | NA | NA | 1 | 0.9985816 | NA | NA | NA | 0.00 | 15.0 | 170.0 | 18.4772727 | 24.7586253 | NA | NA | ▇▁▁▁▁ |
| ti\_prev\_1to5y | Trachomatous Inflammation-Intense (TI) Prevalence (%) among 1-5 year olds | numeric | NA | NA | 248 | 0.6482270 | NA | NA | NA | 0.00 | 0.0 | 100.0 | 12.5760842 | 18.8374967 | NA | NA | ▇▂▁▁▁ |
| pgp3\_n\_pos\_1to5y | No. of Pgp3 positives among 1-5 year olds | numeric | NA | NA | 1 | 0.9985816 | NA | NA | NA | 0.00 | 1.0 | 54.0 | 4.8323864 | 7.3984564 | NA | NA | ▇▂▁▁▁ |
| pgp3\_n\_tested\_1to5y | No. of 1-5 year olds tested for Pgp3 | numeric | NA | NA | 1 | 0.9985816 | NA | NA | NA | 1.00 | 27.0 | 170.0 | 29.7556818 | 19.4154980 | NA | NA | ▇▂▁▁▁ |
| pgp3\_mean\_1to5y | Mean Pgp3 IgG levels among 1-5 year olds | numeric | NA | NA | 145 | 0.7943262 | NA | NA | NA | 0.00 | 1340.7 | 24866.3 | 4352.2609194 | 5658.4410594 | NA | NA | ▇▂▂▁▁ |
| pgp3\_log10mean\_1to5y | Mean Pgp3 IgG levels (log10) among 1-5 year olds | numeric | NA | NA | 145 | 0.7943262 | NA | NA | NA | 0.00 | 1.1 | 4.1 | 1.4427669 | 0.8749414 | NA | NA | ▇▇▅▃▁ |
| pgp3\_log10geomean\_1to5y | 10^pgp3\_n\_log10mean\_1to5y | numeric | NA | NA | 145 | 0.7943262 | NA | NA | NA | 1.00 | 11.9 | 12403.9 | 273.9216698 | 960.4724795 | NA | NA | ▇▁▁▁▁ |
| pgp3\_prev\_1to5y | Pgp3 IgG seroprevalence (%) among 1-5 year olds | numeric | NA | NA | 1 | 0.9985816 | NA | NA | NA | 0.00 | 5.7 | 93.1 | 16.1309019 | 20.6598982 | NA | NA | ▇▂▂▁▁ |
| ct694\_n\_pos\_1to5y | No. of CT694 seropositives among 1-5 year olds | numeric | NA | NA | 1 | 0.9985816 | NA | NA | NA | 0.00 | 1.0 | 61.0 | 4.0156250 | 7.1052670 | NA | NA | ▇▁▁▁▁ |
| ct694\_n\_tested\_1to5y | No. of 1-5 year olds tested for CT694 | numeric | NA | NA | 1 | 0.9985816 | NA | NA | NA | 0.00 | 21.0 | 170.0 | 22.6193182 | 23.5183406 | NA | NA | ▇▂▁▁▁ |
| ct694\_mean\_1to5y | Mean CT694 IgG levels among 1-5 year olds | numeric | NA | NA | 195 | 0.7234043 | NA | NA | NA | 1.50 | 340.0 | 11021.5 | 1738.8930679 | 2379.7036451 | NA | NA | ▇▂▁▁▁ |
| ct694\_log10mean\_1to5y | Mean CT694 IgG levels (log10) among 1-5 year olds | numeric | NA | NA | 195 | 0.7234043 | NA | NA | NA | 0.15 | 1.4 | 3.5 | 1.5542681 | 0.6577118 | NA | NA | ▂▇▅▃▁ |
| ct694\_log10geomean\_1to5y | 10^ct694\_log10mean\_1to5y | numeric | NA | NA | 195 | 0.7234043 | NA | NA | NA | 1.41 | 27.5 | 2900.6 | 124.7837485 | 294.2881345 | NA | NA | ▇▁▁▁▁ |
| ct694\_prev\_1to5y | CT694 IgG seroprevalence (%) among 1-5 year olds | numeric | NA | NA | 195 | 0.7234043 | NA | NA | NA | 0.00 | 8.7 | 83.3 | 17.6580900 | 19.8562296 | NA | NA | ▇▃▂▁▁ |
| pgp3ct694\_n\_pos\_1to5y | No. of Pgp3 and CT694 positives among 1-5 year olds | numeric | NA | NA | 1 | 0.9985816 | NA | NA | NA | 0.00 | 0.0 | 51.0 | 3.5596591 | 6.6211754 | NA | NA | ▇▁▁▁▁ |
| pgp3ct694\_n\_tested\_1to5y | No. of 1-5 year olds tested for Pgp3 and CT694 | numeric | NA | NA | 1 | 0.9985816 | NA | NA | NA | 0.00 | 21.0 | 170.0 | 22.6193182 | 23.5183406 | NA | NA | ▇▂▁▁▁ |
| pgp3ct694\_prev\_1to5y | Pgp3 and CT694 (+ to both) IgG seroprevalence (%) among 1-5 year olds | numeric | NA | NA | 195 | 0.7234043 | NA | NA | NA | 0.00 | 5.7 | 80.0 | 16.0409332 | 20.0139015 | NA | NA | ▇▂▂▁▁ |

JSON-LD metadata The following JSON-LD can be found by search engines, if you share this codebook publicly on the web.

```
{
  "name": "trachoma_serology_public_data_cluster_v2",
  "description": "This is a codebook for the trachoma_serology_public_data_cluster_v2 (cluster level).\n\n\n## Table of variables\nThis table contains variable names, labels, and number of missing values.\nSee the complete codebook for more.\n\n[truncated]\n\n### Note\nThis dataset was automatically described using the [codebook R package](https://rubenarslan.github.io/codebook/) (version 0.9.2).",
  "datePublished": "2023-04-07",
  "keywords": ["study_id", "cluster_id_public", "country", "district", "eu", "eu_desc", "year", "location_year_name", "mda", "pcr_n_pos", "pcr_n_tested", "tf_n_pos", "tf_n_tested", "ti_n_pos", "ti_n_tested", "pgp3_n_pos", "pgp3_n_tested", "ct694_n_pos", "ct694_n_tested", "pgp3ct694_n_pos", "pgp3ct694_n_tested", "pgp3_mean", "pgp3_log10mean", "pgp3_log10geomean", "ct694_mean", "ct694_log10mean", "ct694_log10geomean", "pcr_prev", "tf_prev", "ti_prev", "pgp3_prev", "ct694_prev", "pgp3ct694_prev", "pcr_n_pos_1to5y", "pcr_n_tested_1to5y", "pcr_prev_1to5y", "tf_n_pos_1to5y", "tf_n_tested_1to5y", "tf_prev_1to5y", "ti_n_pos_1to5y", "ti_n_tested_1to5y", "ti_prev_1to5y", "pgp3_n_pos_1to5y", "pgp3_n_tested_1to5y", "pgp3_mean_1to5y", "pgp3_log10mean_1to5y", "pgp3_log10geomean_1to5y", "pgp3_prev_1to5y", "ct694_n_pos_1to5y", "ct694_n_tested_1to5y", "ct694_mean_1to5y", "ct694_log10mean_1to5y", "ct694_log10geomean_1to5y", "ct694_prev_1to5y", "pgp3ct694_n_pos_1to5y", "pgp3ct694_n_tested_1to5y", "pgp3ct694_prev_1to5y"],
  "@context": "http://schema.org/",
  "@type": "Dataset",
  "variableMeasured": [
    {
      "name": "study_id",
      "description": "Study ID",
      "@type": "propertyValue"
    },
    {
      "name": "cluster_id_public",
      "description": "Public cluster ID",
      "@type": "propertyValue"
    },
    {
      "name": "country",
      "description": "Country",
      "@type": "propertyValue"
    },
    {
      "name": "district",
      "description": "District",
      "@type": "propertyValue"
    },
    {
      "name": "eu",
      "description": "Evaluation unit",
      "@type": "propertyValue"
    },
    {
      "name": "eu_desc",
      "description": "Evaluation unit description",
      "@type": "propertyValue"
    },
    {
      "name": "year",
      "description": "Survey year",
      "@type": "propertyValue"
    },
    {
      "name": "location_year_name",
      "description": "Location and year",
      "value": "1. Agdaz, Morocco 2019,\n2. Alefa, Ethiopia 2017,\n3. Andabet, Ethiopia 2017,\n4. Boumalne Dades, Morocco 2019,\n5. Chikwawa, Malawi 2014,\n6. Dera, Ethiopia 2017,\n7. Dosso, Niger (MORDOR) 2015,\n8. Dosso, Niger (MORDOR) 2016,\n9. Dosso, Niger (MORDOR) 2017,\n10. Dosso, Niger (MORDOR) 2018,\n11. Kongwa, Tanzania 2013,\n12. Kongwa, Tanzania 2014,\n13. Kongwa, Tanzania 2015,\n14. Kongwa, Tanzania 2018,\n15. Matameye, Niger (PRET) 2013,\n16. Mchinji, Malawi 2014,\n17. Wag Hemra, Ethiopia (TAITU) 2018,\n18. Wag Hemra, Ethiopia (WUHA) 2016,\n19. Wag Hemra, Ethiopia (WUHA) 2017,\n20. Wag Hemra, Ethiopia (WUHA) 2018,\n21. Wag Hemra, Ethiopia (WUHA) 2019,\n22. Woreta town, Ethiopia 2017",
      "@type": "propertyValue"
    },
    {
      "name": "mda",
      "description": "MDA administration within the previous year",
      "value": "1. Yes,\n0. No",
      "maxValue": 1,
      "minValue": 0,
      "@type": "propertyValue"
    },
    {
      "name": "pcr_n_pos",
      "description": "No. of PCR infections detected",
      "@type": "propertyValue"
    },
    {
      "name": "pcr_n_tested",
      "description": "No. of individuals tested for infection by PCR",
      "@type": "propertyValue"
    },
    {
      "name": "tf_n_pos",
      "description": "No. of individuals positive for TF",
      "@type": "propertyValue"
    },
    {
      "name": "tf_n_tested",
      "description": "No. of individuals tested for TF",
      "@type": "propertyValue"
    },
    {
      "name": "ti_n_pos",
      "description": "No. of individuals positive for TI",
      "@type": "propertyValue"
    },
    {
      "name": "ti_n_tested",
      "description": "No. of individuals tested for TI",
      "@type": "propertyValue"
    },
    {
      "name": "pgp3_n_pos",
      "description": "No. of positive Pgp3 response",
      "@type": "propertyValue"
    },
    {
      "name": "pgp3_n_tested",
      "description": "No. of individuals tested for Pgp3",
      "@type": "propertyValue"
    },
    {
      "name": "ct694_n_pos",
      "description": "No. of positive CT694 response",
      "@type": "propertyValue"
    },
    {
      "name": "ct694_n_tested",
      "description": "No. of individuals tested for CT694",
      "@type": "propertyValue"
    },
    {
      "name": "pgp3ct694_n_pos",
      "description": "No. Pgp3 and CT694 seropositives",
      "@type": "propertyValue"
    },
    {
      "name": "pgp3ct694_n_tested",
      "description": "No. of individuals tested for Pgp3 and CT694",
      "@type": "propertyValue"
    },
    {
      "name": "pgp3_mean",
      "description": "Mean Pgp3 IgG level",
      "@type": "propertyValue"
    },
    {
      "name": "pgp3_log10mean",
      "description": "Mean Pgp3 IgG level (log10)",
      "@type": "propertyValue"
    },
    {
      "name": "pgp3_log10geomean",
      "description": "10^pgp3_log10mean",
      "@type": "propertyValue"
    },
    {
      "name": "ct694_mean",
      "description": "Mean CT694 IgG level",
      "@type": "propertyValue"
    },
    {
      "name": "ct694_log10mean",
      "description": "Mean CT694 IgG level (log10)",
      "@type": "propertyValue"
    },
    {
      "name": "ct694_log10geomean",
      "description": "10^ct694_log10mean",
      "@type": "propertyValue"
    },
    {
      "name": "pcr_prev",
      "description": "PCR infection prevalence (%)",
      "@type": "propertyValue"
    },
    {
      "name": "tf_prev",
      "description": "Trachomatous Inflammation-Follicular (TF) prevalence (%)",
      "@type": "propertyValue"
    },
    {
      "name": "ti_prev",
      "description": "Trachomatous Inflammation-Intense (TI) Prevalence (%)",
      "@type": "propertyValue"
    },
    {
      "name": "pgp3_prev",
      "description": "Pgp3 IgG seroprevalence (%)",
      "@type": "propertyValue"
    },
    {
      "name": "ct694_prev",
      "description": "CT694 IgG seroprevalence (%)",
      "@type": "propertyValue"
    },
    {
      "name": "pgp3ct694_prev",
      "description": "Pgp3 and CT694 (+ to both) IgG seroprevalence (%)",
      "@type": "propertyValue"
    },
    {
      "name": "pcr_n_pos_1to5y",
      "description": "No. of PCR infections detected among 1-5 year olds",
      "@type": "propertyValue"
    },
    {
      "name": "pcr_n_tested_1to5y",
      "description": "No. of 1-5 year olds tested for infection by PCR",
      "@type": "propertyValue"
    },
    {
      "name": "pcr_prev_1to5y",
      "description": "PCR infection prevalence (%) among 1-5 year olds",
      "@type": "propertyValue"
    },
    {
      "name": "tf_n_pos_1to5y",
      "description": "No. of TF positives among 1-5 year olds",
      "@type": "propertyValue"
    },
    {
      "name": "tf_n_tested_1to5y",
      "description": "No. of 1-5 year olds tested for TF",
      "@type": "propertyValue"
    },
    {
      "name": "tf_prev_1to5y",
      "description": "Trachomatous Inflammation-Follicular (TF) prevalence (%) among 1-5 year olds",
      "@type": "propertyValue"
    },
    {
      "name": "ti_n_pos_1to5y",
      "description": "No. of TI positives among 1-5 year olds",
      "@type": "propertyValue"
    },
    {
      "name": "ti_n_tested_1to5y",
      "description": "No. of 1-5 year olds tested for TI",
      "@type": "propertyValue"
    },
    {
      "name": "ti_prev_1to5y",
      "description": "Trachomatous Inflammation-Intense (TI) Prevalence (%) among 1-5 year olds",
      "@type": "propertyValue"
    },
    {
      "name": "pgp3_n_pos_1to5y",
      "description": "No. of Pgp3 positives among 1-5 year olds",
      "@type": "propertyValue"
    },
    {
      "name": "pgp3_n_tested_1to5y",
      "description": "No. of 1-5 year olds tested for Pgp3",
      "@type": "propertyValue"
    },
    {
      "name": "pgp3_mean_1to5y",
      "description": "Mean Pgp3 IgG levels among 1-5 year olds",
      "@type": "propertyValue"
    },
    {
      "name": "pgp3_log10mean_1to5y",
      "description": "Mean Pgp3 IgG levels (log10) among 1-5 year olds",
      "@type": "propertyValue"
    },
    {
      "name": "pgp3_log10geomean_1to5y",
      "description": "10^pgp3_n_log10mean_1to5y",
      "@type": "propertyValue"
    },
    {
      "name": "pgp3_prev_1to5y",
      "description": "Pgp3 IgG seroprevalence (%) among 1-5 year olds",
      "@type": "propertyValue"
    },
    {
      "name": "ct694_n_pos_1to5y",
      "description": "No. of CT694 seropositives among 1-5 year olds",
      "@type": "propertyValue"
    },
    {
      "name": "ct694_n_tested_1to5y",
      "description": "No. of 1-5 year olds tested for CT694",
      "@type": "propertyValue"
    },
    {
      "name": "ct694_mean_1to5y",
      "description": "Mean CT694 IgG levels among 1-5 year olds",
      "@type": "propertyValue"
    },
    {
      "name": "ct694_log10mean_1to5y",
      "description": "Mean CT694 IgG levels (log10) among 1-5 year olds",
      "@type": "propertyValue"
    },
    {
      "name": "ct694_log10geomean_1to5y",
      "description": "10^ct694_log10mean_1to5y",
      "@type": "propertyValue"
    },
    {
      "name": "ct694_prev_1to5y",
      "description": "CT694 IgG seroprevalence (%) among 1-5 year olds",
      "@type": "propertyValue"
    },
    {
      "name": "pgp3ct694_n_pos_1to5y",
      "description": "No. of Pgp3 and CT694 positives among 1-5 year olds",
      "@type": "propertyValue"
    },
    {
      "name": "pgp3ct694_n_tested_1to5y",
      "description": "No. of 1-5 year olds tested for Pgp3 and CT694",
      "@type": "propertyValue"
    },
    {
      "name": "pgp3ct694_prev_1to5y",
      "description": "Pgp3 and CT694 (+ to both) IgG seroprevalence (%) among 1-5 year olds",
      "@type": "propertyValue"
    }
  ]
}`
```
