# Supplementary material for: Monitoring transmission intensity of trachoma with serology
Source: Nat Commun. 2023 Jun 5;14:3269. doi: 10.1038/s41467-023-38940-5 (PMC10241377; doi:10.1038/s41467-023-38940-5)
Supplement: Supplementary file 6 — Source Data [file 41467_2023_38940_MOESM6_ESM.zip › trachoma_serology_public_data_indiv_codebook.html]

Trachoma serology individual-level public data codebook


Code 

- Show All Code
- Hide All Code

# Trachoma serology individual-level public data codebook

#### updated: 2023-04-07 18:25:09

# Summary

Here, you will find the codebook for the file `trachoma_serology_public_data_indiv_v2` (individual-level data). There are two formats for each dataset, comma separated values (`.csv`) and R dataset (`.rds`). The contents are the same, but the `.rds` file includes additional encodings, such as factor labels and levels.

Note that there is a companion dataset with information aggregated to the study cluster level, named `trachoma_serology_public_data_cluster_v2`. The cluster level dataset includes some additional data that were only available at the cluster level (and not at the individual level): PRET2013 (PCR and TF/TI), Kongwa2013 (PCR), and TCC-Ethiopia2017 (TF/TI).

These data were harmonized and made publicly available under the NIH-funded study: *Seroepidemiology of trachoma for the elimination endgame* R01-AI158884.

The R Markdown script that created this codebook is `05-codebook-trachoma-serology-indiv.Rmd` which is called from the R script `05-codebook-trachoma-serology.R`.

The code below is based on Ruben Arslan’s article on the “codebook” package: https://journals.sagepub.com/doi/full/10.1177/2515245919838783.

```
library(here)
source(here("R/0-config.R"))

# individual dataset
codebook_data_indiv <- read_rds(file = paste0(box_data_path, "/public-", data_version, "/trachoma_serology_public_data_indiv_",data_version,".rds"))


# to import an SPSS file from the same folder uncomment and edit the line below
# codebook_data <- rio::import("mydata.sav")
# for Stata
# codebook_data <- rio::import("mydata.dta")
# for CSV
# codebook_data <- rio::import("mydata.csv") (R. Arslan)
```

# trachoma\_serology\_public\_data\_indiv\_v2

```
# "omit the following lines, if your missing values are already properly labelled" (R. Arslan)
codebook_data_indiv <- detect_missing(codebook_data_indiv,
    only_labelled = FALSE, # "only labelled values are autodetected as
                                   # missing
    negative_values_are_missing = FALSE, # negative values are missing values
    ninety_nine_problems = TRUE,   # 99/999 are missing values, if they
                                   # are more than 5 MAD from the median" (R. Arslan)
    )

# labelling variables
var_label(codebook_data_indiv) <- list(study_id = "Study ID", country = "Country", district = "District",
                                       eu = "Evaluation unit", eu_desc = "Type of evaluation unit",
                                       cluster_id_public = "Public cluster ID", 
                                       household_id_public = "Public household ID", 
                                       individual_id_public = "Public individual ID",
                                       survey = "Month of follow-up", year = "Survey year", 
                                       location_name = "Name of location", 
                                       location_year_name = "Location and year", 
                                       mda = "MDA administration within the previous year", 
                                       age_months = "Age in months", 
                                       age_years = "Age in years", #sex = "Child's sex at birth", 
                                       # exluded sex var for now until all coding for each study is confirmed
                                       pcr_desc = "Type of PCR assay used",
                                       pcr = "PCR test result", 
                                       tf_ti_desc = "Clinical grading used to measure TF/TI",
                                       tf = "Presence of Trachomatous Inflammation-Follicular (TF) in at least one eye", ti = "Presence of Trachomatous Inflammation-Intense (TI) in at least one eye", 
                                       serology_desc = "Type of serology assay used to test for Pgp3 and (if measured) CT694",
                                       pgp3_mfi = "Pgp3 IgG levels in MFI-bg (Luminex)", 
                                       pgp3_elisa = "Pgp3 IgG levels in OD units (ELISA)",
                                       pgp3_mfi_nonneg = "Pgp3 (non-negative) MFI-bg IgG values <=0 recoded as 1 to allow log transform",
                                       pgp3_mfi_log10 = "Pgp3 log10 MFI-bg IgG values", 
                                       pgp3_mfi_cutoff = "Pgp3 IgG MFI-bg seropositivy cutoff value",
                                       pgp3_pos = "Pgp3 seropositive (binary) response",
                                       ct694_mfi = "CT694 IgG levels in MFI-bg (Luminex)", 
                                       ct694_mfi_nonneg = "CT694 (non-negative) IgG values <=0 recoded as 1 to allow log transform",
                                       ct694_mfi_log10 = "CT694 log10 MFI-bg IgG values", 
                                       ct694_mfi_cutoff = "CT694 IgG MFI-bg seropositivy cutoff value",
                                       ct694_pos = "CT694 seropositive (binary) response", 
                                       pgp3ct694_pos = "Seropositive to both Pgp3 and CT694 IgG",
                                       PMID = "PubMed ID of the primary study(ies) that generated the data",
                                       public_data = "If the data from this study have been previously published, URL of previously published dataset(s)"
                                 )

# labelling factor levels
val_labels(codebook_data_indiv$tf) <- c("Present" = 1, "Absent" = 0)
val_labels(codebook_data_indiv$ti) <- c("Present" = 1, "Absent" = 0)
val_labels(codebook_data_indiv$pcr) <- c("Positive" = 1, "Negative" = 0)
val_labels(codebook_data_indiv$pgp3_pos) <- c("Seropositive" = 1, "Seronegative" = 0)
val_labels(codebook_data_indiv$ct694_pos) <- c("Seropositive" = 1, "Seronegative" = 0)
val_labels(codebook_data_indiv$pgp3ct694_pos) <- c("Yes" = 1, "No" = 0)

val_labels(codebook_data_indiv$mda) <- c("Yes" = 1, "No" = 0)

# "If you are not using formr, the codebook package needs to guess which items
# form a scale. The following line finds item aggregates with names like this:
# scale = scale_1 + scale_2R + scale_3R
# identifying these aggregates allows the codebook function to
# automatically compute reliabilities.
# However, it will not reverse items automatically." (R. Arslan)
codebook_data_indiv <- detect_scales(codebook_data_indiv)
```

```
## Warning in detect_scales(codebook_data_indiv): pgp3_mfi_log items found, but no
## aggregate
```

```
## Warning in detect_scales(codebook_data_indiv): ct694_mfi_log items found, but no
## aggregate
```

Create codebook

```
metadata(codebook_data_indiv)$name <- "trachoma_serology_public_data_indiv_v2"
metadata(codebook_data_indiv)$description <- "This is a codebook for the trachoma_serology_public_data_indiv_v2 (individual level)."

codebook(codebook_data_indiv)
```

### Metadata

#### Description

**Dataset name**: trachoma\_serology\_public\_data\_indiv\_v2

This is a codebook for the trachoma\_serology\_public\_data\_indiv\_v2 (individual level).

Metadata for search engines

- **Date published**: 2023-04-07

|  |  |  |  |  |  |  |  |  |  |  |  |  |  |  |  |  |  |  |  |  |  |  |  |  |  |  |  |  |  |  |  |  |  |  |  |  |
| --- | --- | --- | --- | --- | --- | --- | --- | --- | --- | --- | --- | --- | --- | --- | --- | --- | --- | --- | --- | --- | --- | --- | --- | --- | --- | --- | --- | --- | --- | --- | --- | --- | --- | --- | --- | --- |
| | x | | --- | | study\_id | | cluster\_id\_public | | household\_id\_public | | individual\_id\_public | | country | | district | | eu | | eu\_desc | | year | | survey | | mda | | location\_name | | location\_year\_name | | age\_months | | age\_years | | pcr\_desc | | pcr | | tf\_ti\_desc | | tf | | ti | | serology\_desc | | pgp3\_mfi | | pgp3\_mfi\_nonneg | | pgp3\_mfi\_log10 | | pgp3\_elisa | | pgp3\_mfi\_cutoff | | pgp3\_pos | | ct694\_mfi | | ct694\_mfi\_nonneg | | ct694\_mfi\_log10 | | ct694\_mfi\_cutoff | | ct694\_pos | | pgp3ct694\_pos | | PMID | | public\_data | |

#Variables

### study\_id

Study ID

#### Distribution

Distribution of values for study\_id

0 missing values.

#### Summary statistics

| name | label | data\_type | n\_missing | complete\_rate | n\_unique | empty | min | max | whitespace |
| --- | --- | --- | --- | --- | --- | --- | --- | --- | --- |
| study\_id | Study ID | character | 0 | 1 | 9 | 0 | 8 | 16 | 0 |

### cluster\_id\_public

Public cluster ID

#### Distribution

Distribution of values for cluster\_id\_public

0 missing values.

#### Summary statistics

| name | label | data\_type | n\_missing | complete\_rate | n\_unique | empty | min | max | whitespace |
| --- | --- | --- | --- | --- | --- | --- | --- | --- | --- |
| cluster\_id\_public | Public cluster ID | character | 0 | 1 | 482 | 0 | 12 | 27 | 0 |

### household\_id\_public

Public household ID

#### Distribution

Distribution of values for household\_id\_public

9724 missing values.

#### Summary statistics

| name | label | data\_type | n\_missing | complete\_rate | n\_unique | empty | min | max | whitespace |
| --- | --- | --- | --- | --- | --- | --- | --- | --- | --- |
| household\_id\_public | Public household ID | character | 9724 | 0.6888619 | 9605 | 0 | 12 | 52 | 0 |

### individual\_id\_public

Public individual ID

#### Distribution

Distribution of values for individual\_id\_public

0 missing values.

#### Summary statistics

| name | label | data\_type | n\_missing | complete\_rate | n\_unique | empty | min | max | whitespace |
| --- | --- | --- | --- | --- | --- | --- | --- | --- | --- |
| individual\_id\_public | Public individual ID | character | 0 | 1 | 27614 | 0 | 14 | 52 | 0 |

### country

Country

#### Distribution

Distribution of values for country

0 missing values.

#### Summary statistics

| name | label | data\_type | n\_missing | complete\_rate | n\_unique | empty | min | max | whitespace |
| --- | --- | --- | --- | --- | --- | --- | --- | --- | --- |
| country | Country | character | 0 | 1 | 5 | 0 | 5 | 8 | 0 |

### district

District

#### Distribution

Distribution of values for district

0 missing values.

#### Summary statistics

| name | label | data\_type | n\_missing | complete\_rate | n\_unique | empty | min | max | whitespace |
| --- | --- | --- | --- | --- | --- | --- | --- | --- | --- |
| district | District | character | 0 | 1 | 12 | 0 | 4 | 16 | 0 |

### eu

Evaluation unit

#### Distribution

Distribution of values for eu

0 missing values.

#### Summary statistics

| name | label | data\_type | n\_missing | complete\_rate | n\_unique | empty | min | max | whitespace |
| --- | --- | --- | --- | --- | --- | --- | --- | --- | --- |
| eu | Evaluation unit | character | 0 | 1 | 16 | 0 | 4 | 14 | 0 |

### eu\_desc

Type of evaluation unit

#### Distribution

Distribution of values for eu\_desc

0 missing values.

#### Summary statistics

| name | label | data\_type | n\_missing | complete\_rate | n\_unique | empty | min | max | whitespace |
| --- | --- | --- | --- | --- | --- | --- | --- | --- | --- |
| eu\_desc | Type of evaluation unit | character | 0 | 1 | 3 | 0 | 3 | 11 | 0 |

### year

Survey year

#### Distribution

Distribution of values for year

0 missing values.

#### Summary statistics

| name | label | data\_type | n\_missing | complete\_rate | n\_unique | empty | min | max | whitespace |
| --- | --- | --- | --- | --- | --- | --- | --- | --- | --- |
| year | Survey year | character | 0 | 1 | 7 | 0 | 4 | 4 | 0 |

### survey

Month of follow-up

#### Distribution

Distribution of values for survey

13148 missing values.

#### Summary statistics

| name | label | data\_type | n\_missing | complete\_rate | min | median | max | mean | sd | hist |
| --- | --- | --- | --- | --- | --- | --- | --- | --- | --- | --- |
| survey | Month of follow-up | numeric | 13148 | 0.5793044 | 0 | 7 | 36 | 13.84336 | 13.28989 | ▇▂▁▃▃ |

### mda

MDA administration within the previous year

#### Distribution

Distribution of values for mda

0 missing values.

#### Summary statistics

| name | label | data\_type | n\_missing | complete\_rate | min | median | max | mean | sd | n\_value\_labels | hist |
| --- | --- | --- | --- | --- | --- | --- | --- | --- | --- | --- | --- |
| mda | MDA administration within the previous year | haven\_labelled | 0 | 1 | 0 | 1 | 1 | 0.6314914 | 0.482408 | 2 | ▅▁▁▁▁▁▁▇ |

#### Value labels

Response choices

| name | value |
| --- | --- |
| Yes | 1 |
| No | 0 |

### location\_name

Name of location

#### Distribution

Distribution of values for location\_name

0 missing values.

#### Summary statistics

| name | label | data\_type | n\_missing | complete\_rate | n\_unique | empty | min | max | whitespace |
| --- | --- | --- | --- | --- | --- | --- | --- | --- | --- |
| location\_name | Name of location | character | 0 | 1 | 13 | 0 | 14 | 27 | 0 |

### location\_year\_name

Location and year

#### Distribution

Distribution of values for location\_year\_name

0 missing values.

#### Summary statistics

| name | label | data\_type | ordered | value\_labels | n\_missing | complete\_rate | n\_unique | top\_counts |
| --- | --- | --- | --- | --- | --- | --- | --- | --- |
| location\_year\_name | Location and year | factor | FALSE | 1. Agdaz, Morocco 2019, 2. Alefa, Ethiopia 2017, 3. Andabet, Ethiopia 2017, 4. Boumalne Dades, Morocco 2019, 5. Chikwawa, Malawi 2014, 6. Dera, Ethiopia 2017, 7. Dosso, Niger (MORDOR) 2015, 8. Dosso, Niger (MORDOR) 2016, 9. Dosso, Niger (MORDOR) 2017, 10. Dosso, Niger (MORDOR) 2018, 11. Kongwa, Tanzania 2013, 12. Kongwa, Tanzania 2014, 13. Kongwa, Tanzania 2015, 14. Kongwa, Tanzania 2018, 15. Matameye, Niger (PRET) 2013, 16. Mchinji, Malawi 2014, 17. Wag Hemra, Ethiopia (TAITU) 2018, 18. Wag Hemra, Ethiopia (WUHA) 2016, 19. Wag Hemra, Ethiopia (WUHA) 2017, 20. Wag Hemra, Ethiopia (WUHA) 2018, 21. Wag Hemra, Ethiopia (WUHA) 2019, 22. Woreta town, Ethiopia 2017 | 0 | 1 | 22 | Mch: 3355, Chi: 2721, Kon: 2393, Wag: 2310 |

### age\_months

Age in months

#### Distribution

Distribution of values for age\_months

18245 missing values.

#### Summary statistics

| name | label | data\_type | n\_missing | complete\_rate | min | median | max | mean | sd | hist |
| --- | --- | --- | --- | --- | --- | --- | --- | --- | --- | --- |
| age\_months | Age in months | numeric | 18245 | 0.416216 | 12 | 47 | 119 | 47.48455 | 24.61981 | ▆▇▃▂▁ |

### age\_years

Age in years

#### Distribution

Distribution of values for age\_years

0 missing values.

#### Summary statistics

| name | label | data\_type | n\_missing | complete\_rate | min | median | max | mean | sd | hist |
| --- | --- | --- | --- | --- | --- | --- | --- | --- | --- | --- |
| age\_years | Age in years | numeric | 0 | 1 | 1 | 4 | 9 | 4.402521 | 2.428988 | ▆▇▃▅▃ |

### pcr\_desc

Type of PCR assay used

#### Distribution

Distribution of values for pcr\_desc

7318 missing values.

#### Summary statistics

| name | label | data\_type | n\_missing | complete\_rate | n\_unique | empty | min | max | whitespace |
| --- | --- | --- | --- | --- | --- | --- | --- | --- | --- |
| pcr\_desc | Type of PCR assay used | character | 7318 | 0.7658465 | 5 | 0 | 9 | 18 | 0 |

### pcr

PCR test result

#### Distribution

Distribution of values for pcr

16159 missing values.

#### Summary statistics

| name | label | data\_type | n\_missing | complete\_rate | min | median | max | mean | sd | n\_value\_labels | hist |
| --- | --- | --- | --- | --- | --- | --- | --- | --- | --- | --- | --- |
| pcr | PCR test result | haven\_labelled | 16159 | 0.4829616 | 0 | 0 | 1 | 0.1089175 | 0.3115459 | 2 | ▇▁▁▁▁▁▁▁ |

#### Value labels

Response choices

| name | value |
| --- | --- |
| Positive | 1 |
| Negative | 0 |

### tf\_ti\_desc

Clinical grading used to measure TF/TI

#### Distribution

Distribution of values for tf\_ti\_desc

0 missing values.

#### Summary statistics

| name | label | data\_type | n\_missing | complete\_rate | n\_unique | empty | min | max | whitespace |
| --- | --- | --- | --- | --- | --- | --- | --- | --- | --- |
| tf\_ti\_desc | Clinical grading used to measure TF/TI | character | 0 | 1 | 3 | 0 | 11 | 22 | 0 |

### tf

Presence of Trachomatous Inflammation-Follicular (TF) in at least one eye

#### Distribution

Distribution of values for tf

8481 missing values.

#### Summary statistics

| name | label | data\_type | n\_missing | complete\_rate | min | median | max | mean | sd | n\_value\_labels | hist |
| --- | --- | --- | --- | --- | --- | --- | --- | --- | --- | --- | --- |
| tf | Presence of Trachomatous Inflammation-Follicular (TF) in at least one eye | haven\_labelled | 8481 | 0.7286341 | 0 | 0 | 1 | 0.2143422 | 0.4103742 | 2 | ▇▁▁▁▁▁▁▂ |

#### Value labels

Response choices

| name | value |
| --- | --- |
| Present | 1 |
| Absent | 0 |

### ti

Presence of Trachomatous Inflammation-Intense (TI) in at least one eye

#### Distribution

Distribution of values for ti

13204 missing values.

#### Summary statistics

| name | label | data\_type | n\_missing | complete\_rate | min | median | max | mean | sd | n\_value\_labels | hist |
| --- | --- | --- | --- | --- | --- | --- | --- | --- | --- | --- | --- |
| ti | Presence of Trachomatous Inflammation-Intense (TI) in at least one eye | haven\_labelled | 13204 | 0.5775126 | 0 | 0 | 1 | 0.0689235 | 0.253331 | 2 | ▇▁▁▁▁▁▁▁ |

#### Value labels

Response choices

| name | value |
| --- | --- |
| Present | 1 |
| Absent | 0 |

### serology\_desc

Type of serology assay used to test for Pgp3 and (if measured) CT694

#### Distribution

Distribution of values for serology\_desc

0 missing values.

#### Summary statistics

| name | label | data\_type | n\_missing | complete\_rate | n\_unique | empty | min | max | whitespace |
| --- | --- | --- | --- | --- | --- | --- | --- | --- | --- |
| serology\_desc | Type of serology assay used to test for Pgp3 and (if measured) CT694 | character | 0 | 1 | 2 | 0 | 5 | 7 | 0 |

### pgp3\_mfi

Pgp3 IgG levels in MFI-bg (Luminex)

#### Distribution

Distribution of values for pgp3\_mfi

6530 missing values.

#### Summary statistics

| name | label | data\_type | n\_missing | complete\_rate | min | median | max | mean | sd | hist |
| --- | --- | --- | --- | --- | --- | --- | --- | --- | --- | --- |
| pgp3\_mfi | Pgp3 IgG levels in MFI-bg (Luminex) | numeric | 6530 | 0.7910601 | -17 | 9 | 32734 | 5129.759 | 10562.87 | ▇▁▁▁▁ |

### pgp3\_mfi\_nonneg

Pgp3 (non-negative) MFI-bg IgG values <=0 recoded as 1 to allow log transform

#### Distribution

Distribution of values for pgp3\_mfi\_nonneg

6530 missing values.

#### Summary statistics

| name | label | data\_type | n\_missing | complete\_rate | min | median | max | mean | sd | hist |
| --- | --- | --- | --- | --- | --- | --- | --- | --- | --- | --- |
| pgp3\_mfi\_nonneg | Pgp3 (non-negative) MFI-bg IgG values <=0 recoded as 1 to allow log transform | numeric | 6530 | 0.7910601 | 1 | 9 | 32734 | 5129.894 | 10562.8 | ▇▁▁▁▁ |

### pgp3\_mfi\_log10

Pgp3 log10 MFI-bg IgG values

#### Distribution

Distribution of values for pgp3\_mfi\_log10

6523 missing values.

#### Summary statistics

| name | label | data\_type | n\_missing | complete\_rate | min | median | max | mean | sd | hist |
| --- | --- | --- | --- | --- | --- | --- | --- | --- | --- | --- |
| pgp3\_mfi\_log10 | Pgp3 log10 MFI-bg IgG values | numeric | 6523 | 0.791284 | 0 | 0.95 | 4.5 | 1.635946 | 1.538239 | ▇▅▁▁▃ |

### pgp3\_elisa

Pgp3 IgG levels in OD units (ELISA)

#### Distribution

Distribution of values for pgp3\_elisa

25336 missing values.

#### Summary statistics

| name | label | data\_type | n\_missing | complete\_rate | min | median | max | mean | sd | hist |
| --- | --- | --- | --- | --- | --- | --- | --- | --- | --- | --- |
| pgp3\_elisa | Pgp3 IgG levels in OD units (ELISA) | numeric | 25336 | 0.1893258 | 0 | 0.24 | 3.5 | 0.3101973 | 0.2974938 | ▇▁▁▁▁ |

### pgp3\_mfi\_cutoff

Pgp3 IgG MFI-bg seropositivy cutoff value

#### Distribution

Distribution of values for pgp3\_mfi\_cutoff

6076 missing values.

#### Summary statistics

| name | label | data\_type | n\_missing | complete\_rate | min | median | max | mean | sd | hist |
| --- | --- | --- | --- | --- | --- | --- | --- | --- | --- | --- |
| pgp3\_mfi\_cutoff | Pgp3 IgG MFI-bg seropositivy cutoff value | numeric | 6076 | 0.8055867 | 882 | 1113 | 1771 | 1322.052 | 311.7347 | ▃▇▁▂▇ |

### pgp3\_pos

Pgp3 seropositive (binary) response

#### Distribution

Distribution of values for pgp3\_pos

606 missing values.

#### Summary statistics

| name | label | data\_type | n\_missing | complete\_rate | min | median | max | mean | sd | n\_value\_labels | hist |
| --- | --- | --- | --- | --- | --- | --- | --- | --- | --- | --- | --- |
| pgp3\_pos | Pgp3 seropositive (binary) response | haven\_labelled | 606 | 0.9806099 | 0 | 0 | 1 | 0.2075244 | 0.4055409 | 2 | ▇▁▁▁▁▁▁▂ |

#### Value labels

Response choices

| name | value |
| --- | --- |
| Seropositive | 1 |
| Seronegative | 0 |

### ct694\_mfi

CT694 IgG levels in MFI-bg (Luminex)

#### Distribution

Distribution of values for ct694\_mfi

8906 missing values.

#### Summary statistics

| name | label | data\_type | n\_missing | complete\_rate | min | median | max | mean | sd | hist |
| --- | --- | --- | --- | --- | --- | --- | --- | --- | --- | --- |
| ct694\_mfi | CT694 IgG levels in MFI-bg (Luminex) | numeric | 8906 | 0.7150354 | -6 | 19 | 32363 | 2234.18 | 6395.738 | ▇▁▁▁▁ |

### ct694\_mfi\_nonneg

CT694 (non-negative) IgG values <=0 recoded as 1 to allow log transform

#### Distribution

Distribution of values for ct694\_mfi\_nonneg

8906 missing values.

#### Summary statistics

| name | label | data\_type | n\_missing | complete\_rate | min | median | max | mean | sd | hist |
| --- | --- | --- | --- | --- | --- | --- | --- | --- | --- | --- |
| ct694\_mfi\_nonneg | CT694 (non-negative) IgG values <=0 recoded as 1 to allow log transform | numeric | 8906 | 0.7150354 | 1 | 19 | 32363 | 2234.239 | 6395.717 | ▇▁▁▁▁ |

### ct694\_mfi\_log10

CT694 log10 MFI-bg IgG values

#### Distribution

Distribution of values for ct694\_mfi\_log10

8906 missing values.

#### Summary statistics

| name | label | data\_type | n\_missing | complete\_rate | min | median | max | mean | sd | hist |
| --- | --- | --- | --- | --- | --- | --- | --- | --- | --- | --- |
| ct694\_mfi\_log10 | CT694 log10 MFI-bg IgG values | numeric | 8906 | 0.7150354 | 0 | 1.3 | 4.5 | 1.68051 | 1.191954 | ▅▇▂▂▂ |

### ct694\_mfi\_cutoff

CT694 IgG MFI-bg seropositivy cutoff value

#### Distribution

Distribution of values for ct694\_mfi\_cutoff

8469 missing values.

#### Summary statistics

| name | label | data\_type | n\_missing | complete\_rate | min | median | max | mean | sd | hist |
| --- | --- | --- | --- | --- | --- | --- | --- | --- | --- | --- |
| ct694\_mfi\_cutoff | CT694 IgG MFI-bg seropositivy cutoff value | numeric | 8469 | 0.729018 | 137 | 337 | 496 | 318.819 | 118.0509 | ▆▁▇▂▆ |

### ct694\_pos

CT694 seropositive (binary) response

#### Distribution

Distribution of values for ct694\_pos

8906 missing values.

#### Summary statistics

| name | label | data\_type | n\_missing | complete\_rate | min | median | max | mean | sd | n\_value\_labels | hist |
| --- | --- | --- | --- | --- | --- | --- | --- | --- | --- | --- | --- |
| ct694\_pos | CT694 seropositive (binary) response | haven\_labelled | 8906 | 0.7150354 | 0 | 0 | 1 | 0.221193 | 0.4150595 | 2 | ▇▁▁▁▁▁▁▂ |

#### Value labels

Response choices

| name | value |
| --- | --- |
| Seropositive | 1 |
| Seronegative | 0 |

### pgp3ct694\_pos

Seropositive to both Pgp3 and CT694 IgG

#### Distribution

Distribution of values for pgp3ct694\_pos

8906 missing values.

#### Summary statistics

| name | label | data\_type | n\_missing | complete\_rate | min | median | max | mean | sd | n\_value\_labels | hist |
| --- | --- | --- | --- | --- | --- | --- | --- | --- | --- | --- | --- |
| pgp3ct694\_pos | Seropositive to both Pgp3 and CT694 IgG | haven\_labelled | 8906 | 0.7150354 | 0 | 0 | 1 | 0.2012798 | 0.4009656 | 2 | ▇▁▁▁▁▁▁▂ |

#### Value labels

Response choices

| name | value |
| --- | --- |
| Yes | 1 |
| No | 0 |

### PMID

PubMed ID of the primary study(ies) that generated the data

#### Distribution

Distribution of values for PMID

0 missing values.

#### Summary statistics

| name | label | data\_type | n\_missing | complete\_rate | n\_unique | empty | min | max | whitespace |
| --- | --- | --- | --- | --- | --- | --- | --- | --- | --- |
| PMID | PubMed ID of the primary study(ies) that generated the data | character | 0 | 1 | 9 | 0 | 8 | 41 | 0 |

### public\_data

If the data from this study have been previously published, URL of previously published dataset(s)

#### Distribution

Distribution of values for public\_data

13863 missing values.

#### Summary statistics

| name | label | data\_type | n\_missing | complete\_rate | n\_unique | empty | min | max | whitespace |
| --- | --- | --- | --- | --- | --- | --- | --- | --- | --- |
| public\_data | If the data from this study have been previously published, URL of previously published dataset(s) | character | 13863 | 0.5564266 | 4 | 0 | 21 | 56 | 0 |

## Missingness report

## Codebook table

| name | label | data\_type | ordered | value\_labels | n\_missing | complete\_rate | n\_unique | empty | top\_counts | min | median | max | mean | sd | whitespace | n\_value\_labels | hist |
| --- | --- | --- | --- | --- | --- | --- | --- | --- | --- | --- | --- | --- | --- | --- | --- | --- | --- |
| study\_id | Study ID | character | NA | NA | 0 | 1.0000000 | 9 | 0 | NA | 8 | NA | 16 | NA | NA | 0 | NA | NA |
| cluster\_id\_public | Public cluster ID | character | NA | NA | 0 | 1.0000000 | 482 | 0 | NA | 12 | NA | 27 | NA | NA | 0 | NA | NA |
| household\_id\_public | Public household ID | character | NA | NA | 9724 | 0.6888619 | 9605 | 0 | NA | 12 | NA | 52 | NA | NA | 0 | NA | NA |
| individual\_id\_public | Public individual ID | character | NA | NA | 0 | 1.0000000 | 27614 | 0 | NA | 14 | NA | 52 | NA | NA | 0 | NA | NA |
| country | Country | character | NA | NA | 0 | 1.0000000 | 5 | 0 | NA | 5 | NA | 8 | NA | NA | 0 | NA | NA |
| district | District | character | NA | NA | 0 | 1.0000000 | 12 | 0 | NA | 4 | NA | 16 | NA | NA | 0 | NA | NA |
| eu | Evaluation unit | character | NA | NA | 0 | 1.0000000 | 16 | 0 | NA | 4 | NA | 14 | NA | NA | 0 | NA | NA |
| eu\_desc | Type of evaluation unit | character | NA | NA | 0 | 1.0000000 | 3 | 0 | NA | 3 | NA | 11 | NA | NA | 0 | NA | NA |
| year | Survey year | character | NA | NA | 0 | 1.0000000 | 7 | 0 | NA | 4 | NA | 4 | NA | NA | 0 | NA | NA |
| survey | Month of follow-up | numeric | NA | NA | 13148 | 0.5793044 | NA | NA | NA | 0 | 7.00 | 36.0 | 13.8433582 | 1.328989e+01 | NA | NA | ▇▂▁▃▃ |
| mda | MDA administration within the previous year | haven\_labelled | NA | 1. Yes, 0. No | 0 | 1.0000000 | NA | NA | NA | 0 | 1 | 1 | 0.6314914 | 4.824080e-01 | NA | 2 | ▅▁▁▁▁▁▁▇ |
| location\_name | Name of location | character | NA | NA | 0 | 1.0000000 | 13 | 0 | NA | 14 | NA | 27 | NA | NA | 0 | NA | NA |
| location\_year\_name | Location and year | factor | FALSE | 1. Agdaz, Morocco 2019, 2. Alefa, Ethiopia 2017, 3. Andabet, Ethiopia 2017, 4. Boumalne Dades, Morocco 2019, 5. Chikwawa, Malawi 2014, 6. Dera, Ethiopia 2017, 7. Dosso, Niger (MORDOR) 2015, 8. Dosso, Niger (MORDOR) 2016, 9. Dosso, Niger (MORDOR) 2017, 10. Dosso, Niger (MORDOR) 2018, 11. Kongwa, Tanzania 2013, 12. Kongwa, Tanzania 2014, 13. Kongwa, Tanzania 2015, 14. Kongwa, Tanzania 2018, 15. Matameye, Niger (PRET) 2013, 16. Mchinji, Malawi 2014, 17. Wag Hemra, Ethiopia (TAITU) 2018, 18. Wag Hemra, Ethiopia (WUHA) 2016, 19. Wag Hemra, Ethiopia (WUHA) 2017, 20. Wag Hemra, Ethiopia (WUHA) 2018, 21. Wag Hemra, Ethiopia (WUHA) 2019, 22. Woreta town, Ethiopia 2017 | 0 | 1.0000000 | 22 | NA | Mch: 3355, Chi: 2721, Kon: 2393, Wag: 2310 | NA | NA | NA | NA | NA | NA | NA | NA |
| age\_months | Age in months | numeric | NA | NA | 18245 | 0.4162160 | NA | NA | NA | 12 | 47.00 | 119.0 | 47.4845480 | 2.461981e+01 | NA | NA | ▆▇▃▂▁ |
| age\_years | Age in years | numeric | NA | NA | 0 | 1.0000000 | NA | NA | NA | 1 | 4.00 | 9.0 | 4.4025214 | 2.428988e+00 | NA | NA | ▆▇▃▅▃ |
| pcr\_desc | Type of PCR assay used | character | NA | NA | 7318 | 0.7658465 | 5 | 0 | NA | 9 | NA | 18 | NA | NA | 0 | NA | NA |
| pcr | PCR test result | haven\_labelled | NA | 1. Positive, 0. Negative | 16159 | 0.4829616 | NA | NA | NA | 0 | 0 | 1 | 0.1089175 | 3.115459e-01 | NA | 2 | ▇▁▁▁▁▁▁▁ |
| tf\_ti\_desc | Clinical grading used to measure TF/TI | character | NA | NA | 0 | 1.0000000 | 3 | 0 | NA | 11 | NA | 22 | NA | NA | 0 | NA | NA |
| tf | Presence of Trachomatous Inflammation-Follicular (TF) in at least one eye | haven\_labelled | NA | 1. Present, 0. Absent | 8481 | 0.7286341 | NA | NA | NA | 0 | 0 | 1 | 0.2143422 | 4.103742e-01 | NA | 2 | ▇▁▁▁▁▁▁▂ |
| ti | Presence of Trachomatous Inflammation-Intense (TI) in at least one eye | haven\_labelled | NA | 1. Present, 0. Absent | 13204 | 0.5775126 | NA | NA | NA | 0 | 0 | 1 | 0.0689235 | 2.533310e-01 | NA | 2 | ▇▁▁▁▁▁▁▁ |
| serology\_desc | Type of serology assay used to test for Pgp3 and (if measured) CT694 | character | NA | NA | 0 | 1.0000000 | 2 | 0 | NA | 5 | NA | 7 | NA | NA | 0 | NA | NA |
| pgp3\_mfi | Pgp3 IgG levels in MFI-bg (Luminex) | numeric | NA | NA | 6530 | 0.7910601 | NA | NA | NA | -17 | 9.00 | 32734.0 | 5129.7586862 | 1.056287e+04 | NA | NA | ▇▁▁▁▁ |
| pgp3\_mfi\_nonneg | Pgp3 (non-negative) MFI-bg IgG values <=0 recoded as 1 to allow log transform | numeric | NA | NA | 6530 | 0.7910601 | NA | NA | NA | 1 | 9.00 | 32734.0 | 5129.8942280 | 1.056280e+04 | NA | NA | ▇▁▁▁▁ |
| pgp3\_mfi\_log10 | Pgp3 log10 MFI-bg IgG values | numeric | NA | NA | 6523 | 0.7912840 | NA | NA | NA | 0 | 0.95 | 4.5 | 1.6359456 | 1.538239e+00 | NA | NA | ▇▅▁▁▃ |
| pgp3\_elisa | Pgp3 IgG levels in OD units (ELISA) | numeric | NA | NA | 25336 | 0.1893258 | NA | NA | NA | 0 | 0.24 | 3.5 | 0.3101973 | 2.974938e-01 | NA | NA | ▇▁▁▁▁ |
| pgp3\_mfi\_cutoff | Pgp3 IgG MFI-bg seropositivy cutoff value | numeric | NA | NA | 6076 | 0.8055867 | NA | NA | NA | 882 | 1113.00 | 1771.0 | 1322.0517536 | 3.117347e+02 | NA | NA | ▃▇▁▂▇ |
| pgp3\_pos | Pgp3 seropositive (binary) response | haven\_labelled | NA | 1. Seropositive, 0. Seronegative | 606 | 0.9806099 | NA | NA | NA | 0 | 0 | 1 | 0.2075244 | 4.055409e-01 | NA | 2 | ▇▁▁▁▁▁▁▂ |
| ct694\_mfi | CT694 IgG levels in MFI-bg (Luminex) | numeric | NA | NA | 8906 | 0.7150354 | NA | NA | NA | -6 | 19.00 | 32363.0 | 2234.1799347 | 6.395738e+03 | NA | NA | ▇▁▁▁▁ |
| ct694\_mfi\_nonneg | CT694 (non-negative) IgG values <=0 recoded as 1 to allow log transform | numeric | NA | NA | 8906 | 0.7150354 | NA | NA | NA | 1 | 19.00 | 32363.0 | 2234.2387345 | 6.395717e+03 | NA | NA | ▇▁▁▁▁ |
| ct694\_mfi\_log10 | CT694 log10 MFI-bg IgG values | numeric | NA | NA | 8906 | 0.7150354 | NA | NA | NA | 0 | 1.28 | 4.5 | 1.6805102 | 1.191954e+00 | NA | NA | ▅▇▂▂▂ |
| ct694\_mfi\_cutoff | CT694 IgG MFI-bg seropositivy cutoff value | numeric | NA | NA | 8469 | 0.7290180 | NA | NA | NA | 137 | 337.00 | 496.0 | 318.8189519 | 1.180509e+02 | NA | NA | ▆▁▇▂▆ |
| ct694\_pos | CT694 seropositive (binary) response | haven\_labelled | NA | 1. Seropositive, 0. Seronegative | 8906 | 0.7150354 | NA | NA | NA | 0 | 0 | 1 | 0.2211930 | 4.150595e-01 | NA | 2 | ▇▁▁▁▁▁▁▂ |
| pgp3ct694\_pos | Seropositive to both Pgp3 and CT694 IgG | haven\_labelled | NA | 1. Yes, 0. No | 8906 | 0.7150354 | NA | NA | NA | 0 | 0 | 1 | 0.2012798 | 4.009656e-01 | NA | 2 | ▇▁▁▁▁▁▁▂ |
| PMID | PubMed ID of the primary study(ies) that generated the data | character | NA | NA | 0 | 1.0000000 | 9 | 0 | NA | 8 | NA | 41 | NA | NA | 0 | NA | NA |
| public\_data | If the data from this study have been previously published, URL of previously published dataset(s) | character | NA | NA | 13863 | 0.5564266 | 4 | 0 | NA | 21 | NA | 56 | NA | NA | 0 | NA | NA |

JSON-LD metadata The following JSON-LD can be found by search engines, if you share this codebook publicly on the web.

```
{
  "name": "trachoma_serology_public_data_indiv_v2",
  "description": "This is a codebook for the trachoma_serology_public_data_indiv_v2 (individual level).\n\n\n## Table of variables\nThis table contains variable names, labels, and number of missing values.\nSee the complete codebook for more.\n\n[truncated]\n\n### Note\nThis dataset was automatically described using the [codebook R package](https://rubenarslan.github.io/codebook/) (version 0.9.2).",
  "datePublished": "2023-04-07",
  "keywords": ["study_id", "cluster_id_public", "household_id_public", "individual_id_public", "country", "district", "eu", "eu_desc", "year", "survey", "mda", "location_name", "location_year_name", "age_months", "age_years", "pcr_desc", "pcr", "tf_ti_desc", "tf", "ti", "serology_desc", "pgp3_mfi", "pgp3_mfi_nonneg", "pgp3_mfi_log10", "pgp3_elisa", "pgp3_mfi_cutoff", "pgp3_pos", "ct694_mfi", "ct694_mfi_nonneg", "ct694_mfi_log10", "ct694_mfi_cutoff", "ct694_pos", "pgp3ct694_pos", "PMID", "public_data"],
  "@context": "http://schema.org/",
  "@type": "Dataset",
  "variableMeasured": [
    {
      "name": "study_id",
      "description": "Study ID",
      "@type": "propertyValue"
    },
    {
      "name": "cluster_id_public",
      "description": "Public cluster ID",
      "@type": "propertyValue"
    },
    {
      "name": "household_id_public",
      "description": "Public household ID",
      "@type": "propertyValue"
    },
    {
      "name": "individual_id_public",
      "description": "Public individual ID",
      "@type": "propertyValue"
    },
    {
      "name": "country",
      "description": "Country",
      "@type": "propertyValue"
    },
    {
      "name": "district",
      "description": "District",
      "@type": "propertyValue"
    },
    {
      "name": "eu",
      "description": "Evaluation unit",
      "@type": "propertyValue"
    },
    {
      "name": "eu_desc",
      "description": "Type of evaluation unit",
      "@type": "propertyValue"
    },
    {
      "name": "year",
      "description": "Survey year",
      "@type": "propertyValue"
    },
    {
      "name": "survey",
      "description": "Month of follow-up",
      "@type": "propertyValue"
    },
    {
      "name": "mda",
      "description": "MDA administration within the previous year",
      "value": "1. Yes,\n0. No",
      "maxValue": 1,
      "minValue": 0,
      "@type": "propertyValue"
    },
    {
      "name": "location_name",
      "description": "Name of location",
      "@type": "propertyValue"
    },
    {
      "name": "location_year_name",
      "description": "Location and year",
      "value": "1. Agdaz, Morocco 2019,\n2. Alefa, Ethiopia 2017,\n3. Andabet, Ethiopia 2017,\n4. Boumalne Dades, Morocco 2019,\n5. Chikwawa, Malawi 2014,\n6. Dera, Ethiopia 2017,\n7. Dosso, Niger (MORDOR) 2015,\n8. Dosso, Niger (MORDOR) 2016,\n9. Dosso, Niger (MORDOR) 2017,\n10. Dosso, Niger (MORDOR) 2018,\n11. Kongwa, Tanzania 2013,\n12. Kongwa, Tanzania 2014,\n13. Kongwa, Tanzania 2015,\n14. Kongwa, Tanzania 2018,\n15. Matameye, Niger (PRET) 2013,\n16. Mchinji, Malawi 2014,\n17. Wag Hemra, Ethiopia (TAITU) 2018,\n18. Wag Hemra, Ethiopia (WUHA) 2016,\n19. Wag Hemra, Ethiopia (WUHA) 2017,\n20. Wag Hemra, Ethiopia (WUHA) 2018,\n21. Wag Hemra, Ethiopia (WUHA) 2019,\n22. Woreta town, Ethiopia 2017",
      "@type": "propertyValue"
    },
    {
      "name": "age_months",
      "description": "Age in months",
      "@type": "propertyValue"
    },
    {
      "name": "age_years",
      "description": "Age in years",
      "@type": "propertyValue"
    },
    {
      "name": "pcr_desc",
      "description": "Type of PCR assay used",
      "@type": "propertyValue"
    },
    {
      "name": "pcr",
      "description": "PCR test result",
      "value": "1. Positive,\n0. Negative",
      "maxValue": 1,
      "minValue": 0,
      "@type": "propertyValue"
    },
    {
      "name": "tf_ti_desc",
      "description": "Clinical grading used to measure TF/TI",
      "@type": "propertyValue"
    },
    {
      "name": "tf",
      "description": "Presence of Trachomatous Inflammation-Follicular (TF) in at least one eye",
      "value": "1. Present,\n0. Absent",
      "maxValue": 1,
      "minValue": 0,
      "@type": "propertyValue"
    },
    {
      "name": "ti",
      "description": "Presence of Trachomatous Inflammation-Intense (TI) in at least one eye",
      "value": "1. Present,\n0. Absent",
      "maxValue": 1,
      "minValue": 0,
      "@type": "propertyValue"
    },
    {
      "name": "serology_desc",
      "description": "Type of serology assay used to test for Pgp3 and (if measured) CT694",
      "@type": "propertyValue"
    },
    {
      "name": "pgp3_mfi",
      "description": "Pgp3 IgG levels in MFI-bg (Luminex)",
      "@type": "propertyValue"
    },
    {
      "name": "pgp3_mfi_nonneg",
      "description": "Pgp3 (non-negative) MFI-bg IgG values <=0 recoded as 1 to allow log transform",
      "@type": "propertyValue"
    },
    {
      "name": "pgp3_mfi_log10",
      "description": "Pgp3 log10 MFI-bg IgG values",
      "@type": "propertyValue"
    },
    {
      "name": "pgp3_elisa",
      "description": "Pgp3 IgG levels in OD units (ELISA)",
      "@type": "propertyValue"
    },
    {
      "name": "pgp3_mfi_cutoff",
      "description": "Pgp3 IgG MFI-bg seropositivy cutoff value",
      "@type": "propertyValue"
    },
    {
      "name": "pgp3_pos",
      "description": "Pgp3 seropositive (binary) response",
      "value": "1. Seropositive,\n0. Seronegative",
      "maxValue": 1,
      "minValue": 0,
      "@type": "propertyValue"
    },
    {
      "name": "ct694_mfi",
      "description": "CT694 IgG levels in MFI-bg (Luminex)",
      "@type": "propertyValue"
    },
    {
      "name": "ct694_mfi_nonneg",
      "description": "CT694 (non-negative) IgG values <=0 recoded as 1 to allow log transform",
      "@type": "propertyValue"
    },
    {
      "name": "ct694_mfi_log10",
      "description": "CT694 log10 MFI-bg IgG values",
      "@type": "propertyValue"
    },
    {
      "name": "ct694_mfi_cutoff",
      "description": "CT694 IgG MFI-bg seropositivy cutoff value",
      "@type": "propertyValue"
    },
    {
      "name": "ct694_pos",
      "description": "CT694 seropositive (binary) response",
      "value": "1. Seropositive,\n0. Seronegative",
      "maxValue": 1,
      "minValue": 0,
      "@type": "propertyValue"
    },
    {
      "name": "pgp3ct694_pos",
      "description": "Seropositive to both Pgp3 and CT694 IgG",
      "value": "1. Yes,\n0. No",
      "maxValue": 1,
      "minValue": 0,
      "@type": "propertyValue"
    },
    {
      "name": "PMID",
      "description": "PubMed ID of the primary study(ies) that generated the data",
      "@type": "propertyValue"
    },
    {
      "name": "public_data",
      "description": "If the data from this study have been previously published, URL of previously published dataset(s)",
      "@type": "propertyValue"
    }
  ]
}`
```
